# The gut microbiota‐aromatic hydrocarbon receptor (AhR) axis mediates the anticolitic effect of polyphenol‐rich extracts from *Sanghuangporus*

**DOI:** 10.1002/imt2.180

**Published:** 2024-03-11

**Authors:** Shi Zhong, Yu‐Qing Sun, Jin‐Xi Huo, Wen‐Yi Xu, Ya‐Nan Yang, Jun‐Bo Yang, Wei‐Jie Wu, Yong‐Xin Liu, Chong‐Ming Wu, You‐Gui Li

**Affiliations:** ^1^ Institute of Sericulture and Tea Zhejiang Academy of Agricultural Sciences Hangzhou China; ^2^ Beijing QuantiHealth Technology Co., Ltd. Beijing China; ^3^ School of Chinese Materia Medica Tianjin University of Traditional Chinese Medicine Tianjin China; ^4^ Shenzhen Branch, Guangdong Laboratory of Lingnan Modern Agriculture Genome Analysis Laboratory of the Ministry of Agriculture and Rural Affairs, Agricultural Genomics Institute at Shenzhen, Chinese Academy of Agricultural Sciences Shenzhen Guangdong China; ^5^ Food Science Institute Zhejiang Academy of Agricultural Sciences Hangzhou China

**Keywords:** 5‐hydroxyindole‐3‐acetic acid, aromatic hydrocarbon receptor, gut microbiota, inflammatory bowel disease, *Sanghuangporus*

## Abstract

Inflammatory bowel disease (IBD) is a significant global health concern. The gut microbiota plays an essential role in the onset and development of IBD. *Sanghuangporus* (SH), a traditional Chinese medicinal mushroom, has excellent anti‐inflammatory effects and is effective at modulating the gut microbiota. Despite these attributes, the specific anticolitic effects of SH and the mechanisms through which the gut microbiota mediates its benefits remain unclear. Herein, we demonstrated that polyphenol‐rich extract from SH effectively alleviated the pathological symptoms of dextran sodium sulfate (DSS)‐induced colitis in mice by modulating the gut microbiota. Treatment with SH distinctly enriched *Alistipes*, especially *Alistipes onderdonkii*, and its metabolite 5‐hydroxyindole‐3‐acetic acid (5HIAA). Oral gavage of live *A. onderdonkii* or 5HIAA potently mitigated DSS‐induced colitis in mice. Moreover, both 5HIAA and SH significantly activated the aromatic hydrocarbon receptor (AhR), and the administration of an AhR antagonist abrogated their protective effects against colitis. These results underscore the potent efficacy of SH in diminishing DSS‐induced colitis through the promotion of *A. onderdonkii* and 5HIAA, ultimately activating AhR signaling. This study unveils potential avenues for developing therapeutic strategies for colitis based on the interplay between SH and the gut microbiota.

## Highlights

- *Sanghuangporus* (SH) polyphenol extract potently alleviated symptoms of colon colitis in dextran sodium sulfate‐induced colitis mice.
- The gut microbiota especially *Alistipes onderdonkii* mediates the anticolitic effect of SH.
- The SH‐enriched microbial metabolite 5‐hydroxyindole‐3‐acetic acid exhibited an anticolitic effect via the activation of the aryl hydrocarbon receptor.
- The “gut microbiota‐metabolites‐signaling pathway” provides an effective perspective for the study of complex Chinese medicine systems.

## Graphical abstract

*Sanghuangporus* is a medical mushroom with potent anticolitic effects. It modulates the gut microbiota and specifically enriches *Alistipes onderdonkii*, which can produce 5‐hydroxyindole‐3‐acetic acid, thus activating the aryl hydrocarbon receptor pathway and ameliorating colitis.

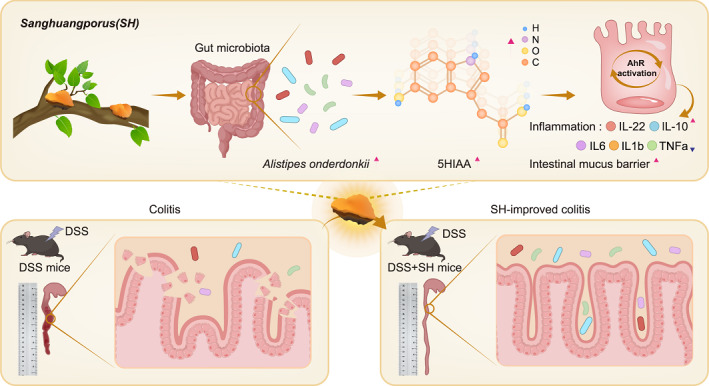

## INTRODUCTION

Inflammatory bowel disease (IBD), which mainly comprises ulcerative colitis (UC) and Crohn's disease, is a worldwide health issue that affects approximately 0.5% of the global population [1]. Typical symptoms of IBD include urgent diarrhea, intermittent abdominal pain, rectal bleeding, and weight loss [2, 3]. In addition to significantly decreasing quality of life, IBD elevates the risk of colon cancer, thereby imposing heavy burdens on individuals and society [1]. To date, IBD lacks a definitive medical cure, and commonly used clinical drugs often result in a high remission rate with subsequent instances of secondary failure [4]. Therefore, new therapeutic interventions that are more effective and safer are urgently needed.

A growing body of evidence underscores the intrinsic association between dysbiosis of the gut microbiota and the onset and progression of IBD [5, 6]. Machiels et al. revealed that dysbiosis in UC patients is characterized by a significant decrease in butyrate‐producing species, such as *Roseburia hominis* and *Faecalibacterium prausnitzii* [7]. Treatment with sodium butyrate can attenuate inflammation and mucosal lesions in colitis [8]. Indole derivatives, important microbial metabolites, have been documented to be beneficial agents for ameliorating experimental UC [9]. For instance, indole‐3‐acetic acid (IAA), indole‐3‐carbinol, and indole‐3‐pyruvic acid can function as natural ligands of the aryl hydrocarbon receptor (AhR) to mitigate IBD by enhancing the serum and tissue levels of anti‐inflammatory interleukins [10, 11, 12, 13, 14]. Hence, the gut microbiota and its microbial metabolites, especially indole derivatives, may represent promising reservoirs for the development of new therapeutic interventions against IBD.

Traditional Chinese medicine (TCM) has been successfully used to treat diseases for thousands of years in China [15]. Mounting evidence highlights the pharmacological benefits of natural medicinal resources [16, 17, 18, 19]. The consumption of medical foods has emerged as a promising approach to disease management. *Sanghuangporus* (SH) is an edible medicinal fungus that is used in both medicine and as a dietary supplement. It has been well documented to possess multiple pharmacological effects, including anti‐inflammatory [20, 21], antitumour [22, 23] and antioxidant effects [24, 25]. Furthermore, it has the capacity to modulate the gut microbiota [26, 27]. However, the therapeutic potential of SH for treating IBDs has not been explored. In this work, we aimed to determine the anticolitic efficacy of a polyphenol‐rich extract of SH and explore whether its beneficial effects are intricately associated with the gut microbiota, as well as the underlying mechanism involved.

In the present study, we first assessed the anticolitic activity of SH and corroborated the critical contribution of the gut microbiota through a comprehensive approach involving in vivo functional verification and fecal microbiota transplantation (FMT). Moreover, we pinpointed the key bacterial species *Alistipes onderdonkii* and its active metabolite 5‐hydroxyindole‐3‐acetic acid (5HIAA) as critical mediators of the colitis‐ameliorating effects of SH. We found that these components exert their influence by activating AhR signaling, thus revealing a microbial mechanism that underlies the anticolitic effects of SH. This study not only contributes to a deeper understanding of the therapeutic potential of SH but also establishes a scientific foundation for future explorations into the therapeutic use of SH and the gut microbiota for managing colitis.

## RESULT

### SH alleviates dextran sodium sulfate (DSS)‐induced colitis in C57BL/6 mice

**Figure 1:**
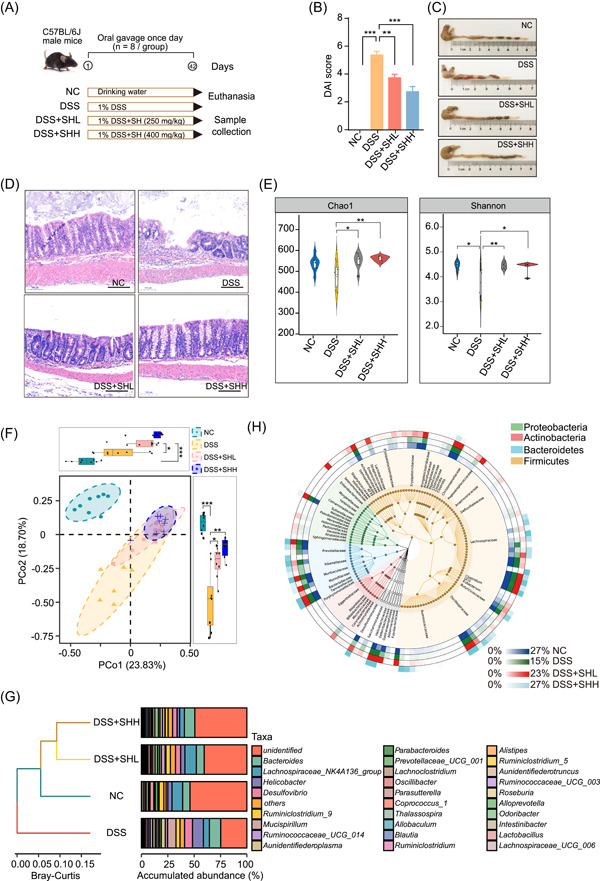
*Sanghuangporus* treatment alleviates DSS‐induced experimental colitis in C57BL/6 mice and alters their gut microbiota. (A) Schematic of the animal experiment. (B) Disease activity index (DAI) score. (C) Representative images of the colonic tissue. (D) Representative images of colonic sections stained with hematoxylin and eosin (scale bars = 50 µm). (E) Alpha diversity of the gut microbiota in each group was assessed by the Chao1 and Shannon indices. (F) Principal coordinate analysis (PCoA) of the gut microbiota in each group based on weighted UniFrac distances. (G) The taxonomic profile of the gut microbiota at the genus level. (H) The core microbiome of DSS‐associated bacteria. The inner ring represents the operational taxonomic units that were reproducibly detected in the NC–DSS–SHL–SHH cohort with the top 150 microbiomes. The relative abundances of distinct microbiomes are shown as blue (NC), green (DSS), red (SHL), and cyan (SHH) heatmaps. The Wilcoxon nonparametric test was used for alpha diversity analysis, and permutational multivariate analysis of variance (PERMANOVA) was used for PCoA analysis. The data are shown as the means ± SEMs (*n* = 8). *p < 0.05; **p < 0.01; ***p < 0.001. DSS, dextran sodium sulfate; NC, negative control; SHH, high dosage of *Sanghuangporus* (400 mg/kg/day); SHL, low dosage of *Sanghuangporus* (250 mg/kg/day).

SH has achieved large‐scale artificial cultivation in China (Figure S1A). SH is a polyphenol‐rich (93.86% ± 2.78%) extract of SH (Figure S1B and Table S1). We first investigated the anticolitic effect of SH in DSS‐induced colitis mice (Figure 1A). As expected, compared with normal mice, the colitis mice exhibited progressive weight loss (Figure S2A), a greater disease activity index (DAI) (Figure 1B), a shorter colon length (Figures 1C and S2B), damaged crypt and colon tissue structures (Figures 1D and S2C), and evident inflammation (including increased tumor necrosis factor‐alpha (TNF‐α), Interleukin‐1beta (IL‐1β), IL‐6, monocyte chemoattractant protein‐1 (MCP‐1), and IL‐17α and decreased IL‐4, IL‐10, and IL‐22) (Figure S3). Both low and high doses of SH ameliorated the pathological symptoms of colitis, as reflected by increased body weight and improved colon length and structure (Figures 1B–D and S2). Furthermore, SH administration reversed the changes in cytokine levels in a dose‐dependent manner (Figure S3), indicating that SH has potent anti‐inflammatory effects.

Oxidative stress and the intestinal mucosal barrier are vital for maintaining permeability to defend against toxins, pathogenic bacteria, and other harmful substances. We then evaluated the protective effect of SH on the expression of the epithelial tight junction (TJ) complex at both the transcriptional and translational levels and detected the expression of oxidative stress‐related genes. Compared with those in the DSS group, the transcription levels of TJ components, such as *Occludin*, *Claudin‐3*, and *Claudin‐4*, were significantly elevated (Figure S4A), and the expression of *NFkappaB*, *Nox4*, and *Stat3* was markedly downregulated in colon tissues (Figure S4B). The expression of TJ proteins was also enhanced by SH (Figure S4C,D), confirming the positive regulatory effect of SH on the mucosal barrier. Moreover, the number of goblet cells also markedly increased after SH treatment (Figure S4E). These results demonstrated that SH significantly ameliorates DSS‐induced colitis in mice.

### The gut microbiota plays a crucial role in mediating the anticolitis efficacy of SH

To evaluate the contribution of the gut microbiota to the anticolitis effect of SH, we performed 16S messenger RNA (rRNA) gene sequencing to assess the impact of SH treatment on the gut microbiota. The α‐diversity of the gut microbiota was obviously lower in DSS‐induced colitis mice than in normal mice (*p* < 0.05). Both low and high dosages of SH supplementation significantly increased the α‐diversity (*p* < 0.05, *p* < 0.01) (Figure 1E). Principal coordinate analysis (PCoA) and hierarchical clustering analysis revealed that SH treatment shifted the gut microbiota toward that of the normal control (NC) (Figure 1F–H). These results indicate that SH can significantly modulate the gut microbial community in DSS‐induced colitis mice.

**Figure 2:**
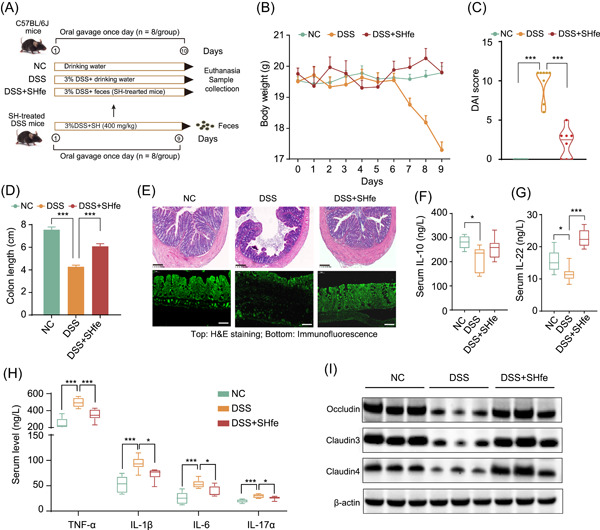
Fecal microbiota transplantation (FMT) reveals the anticolitic effect of the SH‐modulated gut microbiota. (A) Schematic of the animal experiment. (B) Body weight (g) of colitis mice that underwent FMT for 0–9 days. (C, D) Disease activity index (DAI) score (C) and colon length in cm (D) of each group of mice. (E) Representative images of colonic sections stained with hematoxylin and eosin (H&E, top) (scale bars = 200 µm) and immunofluorescence images (bottom) of the structure of tight junctions stained with an antibody against Claudin‐4 (scale bars = 50 µm). (F, G) Serum levels of the anti‐inflammatory cytokines IL‐10 (F) and IL‐22 (G). (H) Serum levels of proinflammatory cytokines (TNF‐α, IL‐1β, IL‐6, and IL‐17α). (I) Western blot analysis of Occludin, Claudin‐3, and Claudin‐4 in colon tissues. Statistical analysis was performed by one‐way analysis of variance (ANOVA) followed by Dunnett's test. The data are shown as the means ± SEMs (*n* = 8). *p < 0.05; **p < 0.01; ***p < 0.001. DSS, dextran sodium sulfate; IL, Interleukin; NC, negative control; SH, *Sanghuangporus*; SHH, high dosage of *Sanghuangporus*; SHL, low dosage of *Sanghuangporus*; TNF‐α, tumor necrosis factor‐alpha.

To further assess whether the SH‐modulated gut microbiota is sufficient to confer anticolitic effects, we established an acute colitis mouse model by supplementing 3% DSS in the drinking water and transplanting feces from SH‐treated mice (donor) to DSS‐induced colitis mice (recipient) (Figure 2A). Because the fecal microbiota from the colitis model mice had no therapeutic effect on colitis (Figure S5), we did not further take this into account in the subsequent analysis. Compared with DSS mice, the recipient mice (DSS+SHfe) showed normal trends in body weight, decreased DAI score, restored colon length, and histology (Figures 2B–E and S6A,B). Fecal transplantation also increased the serum levels of IL‐10 and IL‐22 and decreased the serum levels of TNF‐α, IL‐1β, IL‐6, and IL‐17α (Figure 2F–H), indicating a prominent anti‐inflammatory effect. Importantly, the weakened gut barrier in the colitis mice markedly recovered after transplantation of the SH‐modulated gut microbiota, as reflected by the upregulated expression of *Occludin*, *Claudin‐2*, *Claudin‐3*, and *Claudin‐4* mRNAs and Occludin, Claudin‐3, and Claudin‐4 proteins (Figures 2I and S6C). Therefore, gut microbes from SH‐treated mice exhibited potent colitis‐ameliorating effects.

### SH enriches *A. onderdonkii* to ameliorate colitis

Next, we scrutinized the taxonomic composition of the gut microbiota at the genus level to identify the core bacteria contributing to the anticolitis effect of SH. Comparative analyses were performed between the data from each group and those from the DSS‐induced mice. A total of 12 genera were upregulated, but 25 genera were downregulated in the control, SHL, and SHH groups (Figure S7A). Compared with those in the control group, 34 genera were increased, and 13 genera were decreased. Low‐dose SH treatment induced 10 upregulated genera and 4 downregulated genera. After DSS‐induced mice were administered a high dose of SH, 20 genera were upregulated, and 4 were downregulated (Figure S7B). Differential expression analysis revealed a significant reduction in only *Alistipes* in the DSS group, whereas this change was notably greater after SH treatment (Figure S7C). Further Spearman's correlation analysis indicated that only three genera had negative DAI scores and positive colon length, with *Alistipes* showing the most significant correlation (Figure S7D). These results indicate that SH can significantly modulate the gut microbial community and specifically enrich *Alistipes*.

**Figure 3:**
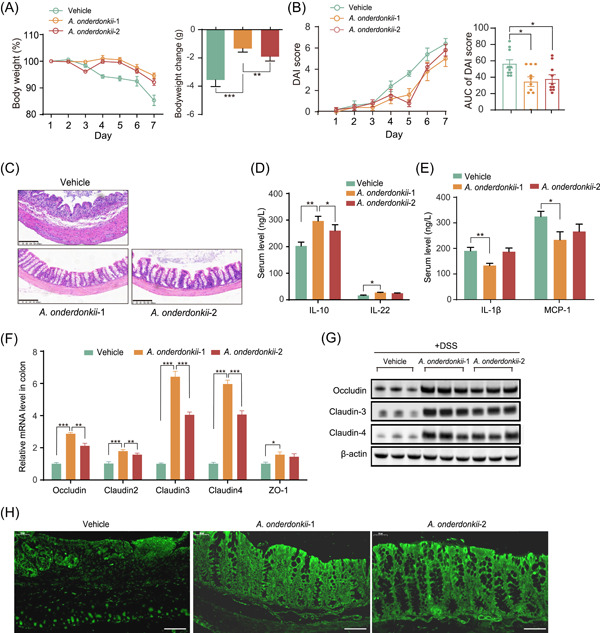
*Alistipes onderdonkii* alleviates DSS‐induced colitis in C57BL/6 mice. (A) Percentage of body weight (%) from 1 to 7 days and change in body weight (g) in each group. (B) DAI score and AUC of the DAI score in each group. (C) Representative images of colonic sections stained with hematoxylin and eosin (scale bars = 200 µm). (D, E) Serum levels of the anti‐inflammatory cytokines IL‐10 and IL‐22 (D) and the proinflammatory cytokines IL‐1β and MCP‐1 (E). (F) Relative mRNA levels of *Occludin*, *Claudin‐2*, *Claudin‐3*, *Claudin‐4*, and *ZO‐1* in colon tissues. (G) Western blot analysis of Occludin, Claudin‐3, and Claudin‐4 expression. (H) Immunofluorescence imaging of tight junction structures using an antibody against Claudin‐4 (scale bars = 50 µm). Statistical analysis was performed by one‐way analysis of variance (ANOVA) followed by Dunnett's test. The data are shown as the means ± SEMs (*n* = 8). *p < 0.05; **p < 0.01; ***p < 0.001. AUC, area under the curve; DAI, disease activity index; DSS, dextran sodium sulfate; HO‐1, heme oxygenase‐1; IL, Interleukin; MCP‐1, monocyte chemoattractant protein‐1; mRNA, messenger RNA.

Furthermore, we quantified fecal *Alistipes* by species‐specific quantitative polymerase chain reaction (qPCR) and found that *A. onderdonkii* was the major *Alistipes* genus enriched in the SH treatment (Figure S7D,E). We obtained three strains of *A. onderdonkii* and evaluated their effects on DSS‐induced colitis. Two of the three *A. onderdonkii* strains (#1: FDB8; #2: FDFM) effectively prevented body weight loss, decreased DAI scores, recovered colon tissue injury, and improved inflammatory status (Figure 3A–E). In addition, *A. onderdonkii* elevated the expression of TJ proteins to enhance gut barrier function (Figure 3F–H). Hence, *A. onderdonkii* could act as a key effective species in conveying the anticolitic effect of SH. Intriguingly, one strain of *A. onderdonkii* (#3) barely improved colitis and even caused harmful effects (Figure S8), exhibiting strain‐specific functions.

### 5HIAA is a key active microbial metabolite

Given the regulatory effect of SH on the gut microbiome, we carried out a metabolomic analysis of fecal materials, aiming to identify functional microbial metabolites. As depicted in Figure S9A, DSS‐induced colitis dramatically altered the metabolites levels compared with those in NC mice (Figure S9A). SH prominently restored the profiles of microbial metabolites, as indicated by the proximity of the microbial metabolites to those in the NC group (Figure S9A). Subsequently, we determined that 5HIAA was significantly elevated by SH treatment (Figure S9B,C). Upon conducting a more comprehensive analysis of the functional gene sequences within the three *Alistipes* strains, it was observed that the genomes of the two *A. onderdonkii* strains (#1: FDB8; #2: FDFM) contained a tpl gene associated with the biosynthesis of indole compounds. In contrast, the genome of the third strain (#3: FDPA) lacked this specific gene (Figure S9D). To prove that *A. onderdonkii* indeed possesses the ability to produce 5HIAA, we detected 5HIAA at concentrations as high as 33.5 μg/mL in the spent culture supernatants of *A. onderdonkii* by high‐performance liquid chromatography (HPLC). Notably, the ability of 5HIAA to produce colitis was correlated with the colitis‐ameliorating effect of *A. onderdonkii*, as the two effective *A. onderdonkii* strains produced more 5HIAA (33.5 and 16.83 μg/mL) than the ineffective strain (0.83 μg/mL) (Figure S9E). Correlation analysis between individual metabolites and colitis indices revealed 22 metabolites strongly associated with colitis symptoms, among which 5HIAA was most strongly positively correlated with colon length and negatively correlated with DAI score (Figure S9F). Therefore, SH could promote the production of 5HIAA, a possible key microbial metabolite associated with the anticolitic effect of SH, in *Alistipes*, especially *A. onderdonkii*.

**Figure 4:**
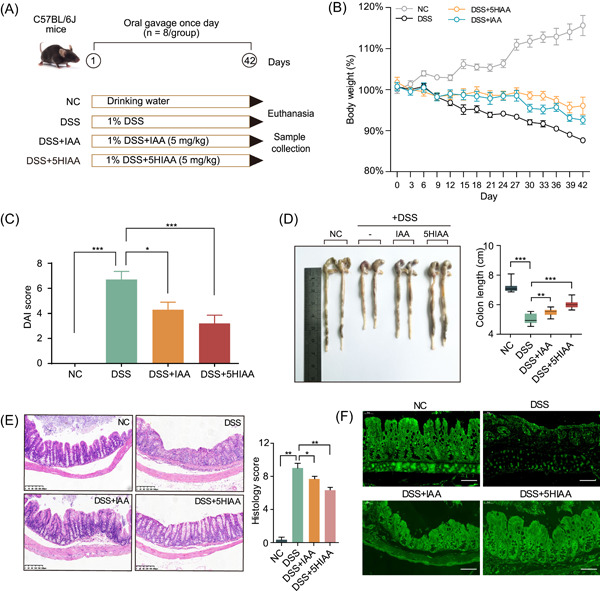
Treatment with 5HIAA alleviates DSS‐induced colitis in C57BL/6 mice. (A) Schematic of the animal experiment. (B) Percentage of body weight (%) from 0 to 42 days. (C) DAI score of each group of mice. (D) Colon length (cm) of each group. (E) Representative images of colonic sections stained with hematoxylin and eosin (H&E) (scale bars = 200 µm) and the histological score of each group of mice. (F) Representative immunofluorescence images of tight junction structures stained with an antibody against Claudin‐4 (scale bars = 50 µm). Statistical analysis was performed by one‐way analysis of variance (ANOVA) followed by Dunnett's test. The data are shown as the means ± SEMs (*n* = 8). *p < 0.05; **p < 0.01; ***p < 0.001. 5HIAA, 5‐hydroxyindole‐3‐acetic acid; DAI, disease activity index; DSS, dextran sodium sulfate; IAA, indole‐3‐acetic acid; NC, negative control.

Gut microbe‐generated IAA has been reported to alleviate colitis [28]; thus, we investigated the effects of 5HIAA, a derivative closely related to IAA, on DSS‐induced colitis (Figure 4A). IAA treatment significantly improved the symptoms of colitis (Figure 4B–F), which was consistent with the findings of previous reports, while 5HIAA performed much better than IAA at alleviating colitis (Figure 4B–F). In addition, both indole derivatives efficiently elevated the levels of anti‐inflammatory factors to attenuate inflammation and decrease the levels of proinflammatory factors (Figure S10A,B). The relative expression of oxidative stress‐related genes (*NFkappaB*, *Nox4*, and *Stat3*) was also reduced by indole derivatives in DSS‐induced mice (Figure S10C). Moreover, the expression of the tight junction factors Occludin and Claudins was upregulated by IAA and 5HIAA, with the latter exhibiting obvious significance (Figure S10D,E).

### Colonic AhR activation is important for mediating the anticolitic effect of SH

**Figure 5:**
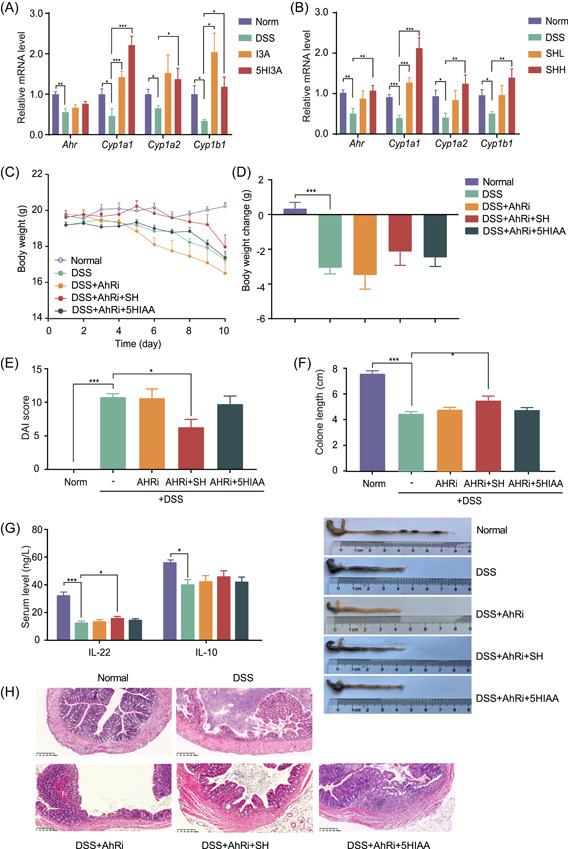
An AhR inhibitor diminishes the anticolitic effects of SH and 5HIAA. (A) Relative mRNA levels of *Ahr*, *Cypa1*, *Cypa2*, and *Cypb1* in the colon tissues of 5HIAA‐treated colitis mice. (B) Relative mRNA levels of *Ahr*, *Cypa1*, *Cypa2*, and *Cypb1* in the colon tissues of SH‐treated colitis mice. (C, D) Body weight (C) of C57BL/6 mice after different treatments from 1 to 10 days and changes in body weight (D) in each group. (E) DAI score. (F) Colon length (cm) of each group of mice. (G) Serum levels of anti‐inflammatory cytokines (IL‐22 and IL‐10). (H) Representative images of colonic tissue and colonic sections stained with hematoxylin and eosin (scale bars = 200 µm). Statistical analysis was performed by one‐way analysis of variance (ANOVA) followed by Dunnett's test. The data are shown as the means ± SEMs (*n* = 8). *p < 0.05; **p < 0.01; ***p < 0.001. 5HIAA, 5‐hydroxyindole‐3‐acetic acid; AhRs, aryl hydrocarbon receptors; DAI, disease activity index; DSS, dextran sodium sulfate; IL, Interleukin; mRNA, messenger RNA; SH, *Sanghuangporus*; SHH, high dosage of *Sanghuangporus*; SHL, low dosage of *Sanghuangporus*.

Previous studies have demonstrated that microbial indole derivatives can protect against colitis by acting as ligands to bind and activate AhR [12, 13], suggesting that SH may improve colitis by enriching *Alistipes* and its metabolite 5HIAA to trigger AhR activation. To confirm this hypothesis, we first examined the colonic expression of genes downstream of AhR (including *Cypa1*, *Cypa2*, and *Cypb1*) in response to 5HIAA and SH. Both treatments significantly upregulated *Cypa1*, *Cypa2*, and *Cypb1* (Figure 5A,B), indicating AhR activation in colon tissues. We then exposed DSS‐treated mice to an AhR inhibitor to verify the contribution of AhR to the anticolitis efficacy of SH. The AhR antagonist StemRegenin 1 essentially abrogated the ameliorative effect of 5HIAA on colitis, as reflected by body weight, DAI, colon length, serum levels of IL‐22 and IL‐10, and colon histology (Figure 5C–H). The beneficial effect of SH treatment on body weight was abolished by the AhR inhibitor (Figure 5C–H), whereas the protective effects on the DAI, colon length, and other indicators were largely diminished by the AhR inhibitor (Figure 5C–H).

The activation of the AhR signaling pathway was further verified through in vitro experiments in Caco‐2 cells. The CCK‐8 assay results demonstrated that all five concentrations of 5HIAA had no cytotoxic effects on Caco‐2 cells (Figure S11A). Although the expression of AhR did not significantly change after 5‐HIAA treatment in Caco‐2 cells, the expression of *Cypa1*, *Cypa2*, and *Cypb1* was notably increased (Figure S11B), suggesting that 5HIAA partially activates the AhR signaling pathway. These results indicate that SH attenuates colitis, at least in most parts, by activating AhR signaling.

## DISCUSSION

IBD poses a significant global public health challenge. Given the adverse effects of clinical drugs and high recurrence rate, the exploration of novel therapeutic strategies for IBD intervention and treatment holds paramount significance [2, 3, 29]. Compelling evidence has demonstrated the intricate connection between the gut microbiota and its metabolites and the development and progression of IBD [5, 9, 30]. Additionally, natural medical resources emerge as a vast potential reservoir, offering effective candidates for managing IBD due to their excellent efficacy and safety, as well as the positive modulation of the gut microbiota [14, 17, 19]. Our study demonstrated the anticolitic effect of SH (a polyphenol‐rich extract from SH, a TCM) and the vital role of the gut microbiota. Briefly, SH‐enriched *A. onderdonkii* promoted the production of its microbial metabolite 5HIAA, which acts as a potent ligand to activate AhR signaling, thereby significantly ameliorating DSS‐induced colitis in mice. This understanding of the microbial mechanism enriches our understanding of the crosstalk between natural medicines and the gut microbiota, facilitating the development of novel therapeutic agents to effectively control colitis.

While the anticolitic effects of SH have been reported in various studies [31, 32], the underlying mechanisms warrant further elucidation. Notably, the beneficial effects of SH on colitis have not been fully elucidated. In recent years, our research team has undertaken systematic investigations into SH, including whole‐genome sequencing. This exploration revealed certain components within SH extracts with anticarcinogenic activity. Moreover, our findings indicate that the mycelia of SH play a regulatory role in growth, immunity, and the fecal microbiota in piglets [33, 34]. In this study, we provided robust evidence confirming for the first time that SH has potent anticolitis efficacy. Notably, we took a significant step forward by revealing the critical role of the gut microbiota in mediating the efficacy of SH. Leveraging FMT, we demonstrated that, compared with DSS‐treated mice, SH‐modulated gut microbiota performed well in ameliorating colitis symptoms, including improvements in body weight, colon length, DAI, inflammatory status, and TJ factors. These results suggest that SH might be a more promising agent for treating colitis by modulating the gut microbiota.

In the intricate landscape of IBD, the consensus is emerging that gut microbial dysbiosis plays a pivotal role in its etiology. Notably, alterations in bacterial taxa and community diversity between IBD patients and healthy individuals have been reported [35]. Specifically, IBD is associated with an increase in the abundance of certain opportunistic pathogens, such as Enterobacteriaceae, and a reduction in the abundance of anti‐inflammatory genera, such as butyrate/propionate producers [35, 36]. We observed marked shifts in the microbial composition of DSS‐treated mice and identified three key genera, namely, *Alistipes*, *Alloprevotella*, and *Clostridium XIVa*, that are negatively associated with colitis symptoms. These genera are SCFA‐producing bacteria that are regarded as beneficial microbes in colitis management [17, 37], indirectly suggesting the beneficial properties of these three genera. Among them, *Alistipes* was the genus most significantly associated with colitis alleviation. There are controversial opinions regarding the effects of the relatively novel genus *Rikenellaceae* on colitis. Several teams have suggested that *Alistipes* can increase susceptibility to colitis in mice by degrading cellulose, thus leading to alterations in the gut immune response and intestinal epithelial cells [37, 38]. Moschen et al. previously reported that *A. finegoldii* activates IL‐6/STAT3 signaling to promote colitis‐associated cancer development [39]. However, most studies prefer to consider *Alistipes*, at least in most species, as an anti‐inflammatory bacteria with colitis‐alleviating effects [19, 40]. For instance, Dziarski et al. demonstrated that oral gavage of *A. finegoldii* effectively attenuated colitis in mice [40]. In our study, the abundance of *Alistipes* was strongly reduced in experimental colitis mice but was significantly increased in SH‐treated mice. At the species level, we found that *A. onderdonkii* was the key species enriched in the SH treatment. Importantly, we obtained three bacterial strains of *A. onderdonkii*, enabling us to perform functional validation of the gut microbiota at the strain level, which was less common in previous similar studies. Oral administration of live *A. onderdonkii* effectively prevented DSS‐induced colitis, confirming the protective effects of *Alistipes* spp. on colitis control. Notably, one of the three *A. onderdonkii* strains barely alleviated colitis, indicating strain specificity. In fact, strain‐level functional variations have been increasingly recognized by researchers [41, 42], highlighting that the pharmacological effects of the gut microbiota should involve specific strain(s) rather than common bacterial species. Another interesting finding in this work was that the impact of DSS on pathology and the gut microbiota was similar at both low (1%) and high (3%) concentrations, suggesting that the acute and chronic DSS‐based colitis models had uniform pathological and gut microbiota structures.

Indole derivatives, a major type of gut microbial metabolite, potently improve colitis and exhibit great potential for colitis management [10, 13, 14, 43]. We found that 5HIAA was prominently elevated by SH treatment and was positively correlated with colitis alleviation. Moreover, we detected a relatively high amount of 5HIAA in the culture media of two effective *A. onderdonkii* strains, thus establishing a link between active metabolites and metabolite‐producing bacteria. In addition, indole and its derivatives are widely regarded as known ligands for the AhR receptor [10, 13, 44], which is an important hub for regulating intestinal immunity and motivating downstream cascades [45, 46]. Accumulating evidence has revealed that AhR can mitigate colitis by decreasing the serum inflammatory cytokines/chemokines (e.g., TNF‐α, IFN‐γ, MCP‐1, and IL‐17) [47], enhancing the expression of IL‐10 and IL‐22 [12, 48], and strengthening the intestinal epithelial cell barrier [11, 14]. Hence, it is plausible that SH promotes 5HIAA generation to activate AhR. As expected, SH/5HIAA significantly elevated the expression of AhR‐targeting genes in the colon, indicating obvious AhR activation. Consistently, the serum levels of proinflammatory factors decreased, and the levels of anti‐inflammatory factors and TJ complexes increased. Furthermore, the introduction of AhR antagonists indicated that AhR signaling plays a crucial role in the anticolitic activity of SH. Notably, the AhR antagonist abrogated the beneficial effects of 5HIAA on colitis but merely attenuated the effects of SH. Considering this difference, we speculated that the ability of SH to alleviate colitis may be attributed to multiple mechanisms, given the various components contained in SH. For example, in our previous study, we found that SH markedly suppressed the phosphorylation of nuclear factor kappa B inhibitor alpha (IκBα) and downregulated the expression of apoptosis‐associated speck‐like protein (ASC3) and caspase‐1 in the colon [49], suggesting that the NF‐κB signaling pathway and NLRP3/caspase‐1 signaling pathway might also participate in the anticolitis effect of SH. Additionally, *Sanghuangporus sanghuang* mycelia can upregulate the Kelch‐like ECH‐associated protein 1 (Keap1)/erythroid 2‐related factor 2 (Nrf2)/heme oxygenase‐1 (HO‐1) pathway to attenuate oxidative stress [20], thereby improving intestinal injury. It must be emphasized that further investigation could enable a comprehensive understanding of other potential mechanisms. Nevertheless, these results corroborate our hypothesis about the regulatory axis of the “SH‐*Alistipes*−5HIAA‐AhR receptor.”

Although our study provides solid evidence that gut microbes and their metabolite 5HIAA play a key role in the anticolitis effect of SH, this study has several limitations. In our experiment, SH was preventively administered DSS. As a complex extract, SH may sequester DSS, thus preventing DSS‐induced colitis. Therefore, the therapeutic effect of SH should be evaluated in the future to eliminate the possibility that SH may prevent colitis by directly interacting with DSS. Furthermore, there must be other mechanisms, such as the NF‐κB and NLRP3/caspase‐1 signaling pathways, by which SH ameliorates colitis. This speculation is based on the following facts: treatment with an AHR antagonist substantially abolishes the beneficial effect of 5HIAA on colitis. However, the anticolitic efficacy of SH was only partially impaired, indicating that the promotion of 5HIAA was not the sole route through which SH diminishes colitis. Moreover, 5HIAA is an indole metabolite produced from the fermentation of tryptophan by the tryptophanase‐expressing gut microbiome. Therefore, multiple bacteria may have the ability to generate 5HIAA. In this work, we discovered an example bacterium, *A. onderdonkii*, which highlights that the gut microbiota can produce indole derivatives, including 5HIAA and IAA, to alleviate colitis. The other bacteria that produce indole and its derivatives need to be further investigated in future studies. Moreover, it remains unknown whether the gut bacterial species/strain *A. onderdonkii* we identified could be developed since a series of investigations, such as colonization assessment, toxicological testing, and further functional verification, have not been conducted. Hence, additional studies are needed to gain thorough insight into the anticolitic effects and underlying mechanisms of action of SH, which will greatly promote the exploitation and application of SH.

## CONCLUSION

**Figure 6:**
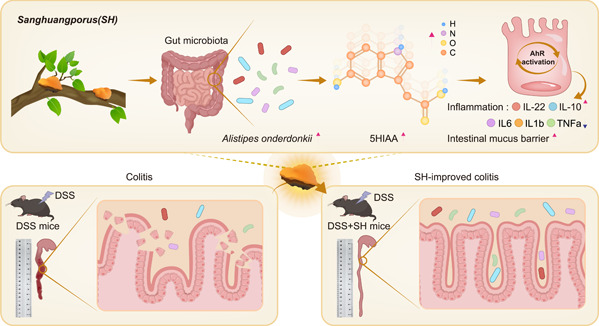
SH alleviates DSS‐induced colitis via the “SH‐gut microbiota‐5HIAA‐AhR axis.” SH enriches the gut microbiota especially *Alistipes onderdonkii* and promotes the generation of gut‐microbiota‐derived metabolites 5HIAA to activate AhR pathway, thus alleviating inflammation and enhancing the intestinal mucus barrier in DSS‐induced colitis mice. 5HIAA, 5‐hydroxyindole‐3‐acetic acid; AhRs, aryl hydrocarbon receptors; DSS, dextran sodium sulfate; IL, Interleukin; SH, *Sanghuangporus*; TNF‐α, tumor necrosis factor‐alpha.

In summary, the TCM SH is effective at improving DSS‐induced colitis through, at least partially, enriching *A. onderdonkii* to elevate the microbial metabolite 5HIAA, thereby activating AhR signaling (Figure 6). The SH‐gut microbiota‐5HIAA‐AhR axis not only indicates a microbial mechanism governing the ability of SH to ameliorate colitis but also provides potential candidates for the development of therapeutic agents to control colitis.

## METHODS

### Study design

The primary objective of our study was to assess the anticolitic effect of a polyphenol‐rich extract of SH, a traditional Chinese medicinal mushroom, and to investigate the contribution of the gut microbiota to mediating the ameliorative efficacy of SH on colitis. Experimental colitis was induced by DSS, and colitis symptoms were evaluated by analyzing body weight changes, colon phenotype and histology, inflammation status, and colon epithelial barrier integrity. Due to the limitations of time, cost and agent amount, the anticolitic effects of different agents were tested in chronic or acute colitis animal models as described in previous studies [50]. The administration of 1%, 2%, or 3% DSS in drinking water reportedly results in the induction of colitis, with 3% DSS leading to the most severe form of colitis. Therefore, a chronic colitis model was established through daily DSS (MP Biologicals) supplementation in drinking water (1.0% w/v) for 42 days to evaluate the anticolitic effects of SH, 5HIAA, and IAA. Acute colitis animal models were established by administering higher concentrations of DSS (such as 3.0% w/v) in the drinking water for a shorter period (such as 10 days) to assess the effects of *A. onderdonkii* and the AhR antagonist. To determine the role of the gut microbiota in alleviating colitis, we conducted an FMT experiment. Specifically, we collected fecal materials from SH‐treated DSS‐treated mice (donors, *n* = 8), pooled the feces, and administered them to another group of DSS‐treated mice (recipients, *n* = 8). Modulation of the microbial community by SH was determined through 16S rRNA gene sequencing and correlation analysis, revealing potential functional bacterial genera/species (*Alistipes* and *A. onderdonkii*). Fecal metabolomic analysis enabled the discovery of active microbial metabolites. Spearman correlation analysis highlighted 5HIAA as a key metabolite, and the connection between *A. onderdonkii* (bacteria) and 5HIAA (metabolite) was confirmed by HPLC analysis.

To validate the colitis‐alleviating effect of *A. onderdonkii* and 5HIAA, DSS‐induced colitis mice were treated with live *A. onderdonkii* (provided by Beijing QuantiHealth Technology Co., Ltd.) or 5HIAA (purchased from Aladdin). Because of the close relationship between 5HIAA and IAA, we proposed that SH‐enriched 5HIAA could activate AhR (acyl hydrocarbon receptor) similar to IAA to mitigate intestinal inflammation and restore the epithelial barrier, thus ameliorating DSS‐induced colitis. To verify this hypothesis, colon tissues from mice administered 5HIAA/SH were examined for the expression of genes downstream of AhR, confirming the activation of colonic AhR by 5HIAA and SH. This pivotal role of AhR was further substantiated by the use of an AhR inhibitor (AhRi) in DSS‐treated mice. Detailed information is listed in the accompanying figure legends and supplemental materials.

### Animal experiments

Animal experiments were approved by the Institutional Animal Care and Use Committee (IACUC) of the Zhejiang Academy of Agricultural Sciences under the number 2021ZAASLA30. All procedures adhered to the Guidelines for the Care and Use of Laboratory Animals issued by the Chinese Council on Animal Research. For all the experiments, 6‐week‐old male C57BL/6 mice were procured from Shanghai Experimental Animal Center (China) and kept under controlled temperature and humidity conditions with a 12 h/12 h light/dark cycle. The rats received normal rodent chow and sterile water.

To assess the anticolitis effect of SH, mice were divided into four groups, each comprising eight mice: the NC group, in which an equal volume of distilled water was administered; the DSS group, in which chronic colitis was induced with daily DSS (MP Biologicals) supplementation in the drinking water (1.0% w/v); and the DSS + SHL and DSS + SHH groups, in which colitis was established with DSS and treated with 250 or 400 mg/kg/day SH, respectively [49]. Body weight was measured every 3 days, and the DAI was evaluated on day 42. At the end of the experiments, the mice were fasted overnight, anaesthetized with sodium pentobarbital and then killed by cervical dislocation for blood collection, colon tissue collection, and fecal sample collection. To determine the serum cytokine levels, the serum was obtained by centrifugation at 4000 rpm for 20 min at 4°C and analyzed using ELISA kits (Thermo Fisher Scientific) following the manufacturer's instructions.

In vivo functional validation of *A. onderdonkii*, 5HIAA, and AhR was carried out according to the experimental design detailed in the supplemental materials.

### Fecal microbiota transplantation

The anticolitic effect of the SH‐modulated gut microbiota was evaluated using FMT in a DSS‐induced acute colitis mouse model. The donor mice were established with colitis by drinking water containing 3.0% (w/v) DSS. Concurrently, the donor mice were gavaged daily with SH (400 mg/kg). Beginning on the seventh day after DSS+SH treatment, fresh fecal materials were obtained from each donor mouse and pooled daily. Approximately 2 g of fresh fecal material from SH‐treated donor mice was suspended in 20 mL of distilled water, filtered through filtration fabric, and centrifuged at 1000 rpm at 4°C. The pellet was washed twice in germ‐free phosphate‐buffered saline (pH 7.0) and resuspended in 2 mL of distilled water to obtain a fecal microbiota solution. Twenty‐four male C57BL/6 mice were randomly divided into three groups (*n* = 8): the NC, DSS, and DSS+SHfe groups. All mice were given a combination of penicillin (2000 U/mL), norfloxacin (3 mg/mL), or streptomycin (2 mg/mL) (Sigma Aldrich) in their drinking water for 5 days to remove indigenous gut microorganisms. The NC group was administered an equal volume of distilled water, while the other two groups developed acute colitis with DSS supplementation in the drinking water (3.0% w/v). The DSS group was gavaged with an equal volume of distilled water, while the DSS+SHfe group was administered 0.2 mL of fecal microbiota solution daily for 9 days, as previously described [51]. Daily assessments included body weight and DAI. At the end of the experiment, blood and colon tissues were collected following the procedures outlined above.

### 16S rRNA gene sequencing

Fecal samples from each mouse were collected, snap‐frozen in liquid nitrogen, and stored at −80°C. Fecal DNA extraction, PCR amplification, and rRNA pyrosequencing were performed by Kaitai Biolab Co., Ltd. following established protocols [18]. Specifically, amplicon library construction and sequencing utilized the Illumina‐compatible bacterial PCR primer pair 319F/806R, which targets the V3–V4 regions of the 16S rRNA gene. Stool DNA was extracted with an E.Z.N.A. Stool DNA Kit (Omega Biotech). PCR was carried out using 2 × Phantamax master mix (Vazyme Biotech) with thermocycling conditions set at 95°C for 1 min, 55°C for 1 min, and 72°C for 1 min for 30 cycles, followed by a final extension at 72°C for 5 min. Triplicate PCRs (50 μL each) were performed and combined after amplification. Before sequencing, the PCR products were extracted using the MiniElute Gel Extraction Kit (QIAGEN) and pooled in equimolar concentrations, and the library's final concentration was determined using a Qubit (Invitrogen). Negative DNA extraction controls (lysis buffer and kit reagents) were also amplified and sequenced to monitor contamination. Sequencing was performed on a NovaSeq instrument (Illumina) using the PE250 strategy.

Quality filtering of the raw tags involved specific filtering conditions to obtain high‐quality clean tags following the Vsearch v2.13.6 quality‐controlled process. Sequencing data clustering was performed with Vsearch v2.13.6, with a similarity level set at 97%. The Classify.seqs command of the Mothur version (1.42.1) software was used to identify the species with the highest similarity to the operational taxonomic unit (OTU) sequence using the RDP annotation database (http://rdp.cme.msu.edu/). Dynamic changes in species diversity among the different groups were calculated using Mothur version (1.42.1) software. OTU abundance information was normalized to a standard sequence number corresponding to the sample with the least number of sequences. Subsequent analyses of alpha and beta diversity were performed based on the output‐normalized data. PCoA was performed using R software, and the data were visualized using the R package [52]. The core bacterial microbiome was visualized using EasyAmplicon [53]. Some bacterial data were visualized using the specialized visualization platform ImageGP [54].

### Genome assembly

The isolated DNA was sequenced on an Illumina HiSeq 2500 platform for short‐read sequencing (150 paired‐end bp) and on a Qitan nanopore for long‐read sequencing [55]. For short‐read sequencing data, adapters were trimmed using Fastp [56] with default parameters. Long‐read sequencing data were subjected to adapter trimming using Porechop (https://github.com/rrwick/Porechop) and filtered by NanoPack [57] with default parameters. All the clean reads were assembled using Unicycler [58] with short and long reads. Genome visualization was achieved through Proksee [59].

### Statistical analysis

The data are expressed as the mean ± SEM and were analyzed using SPSS 17.0, Prism 7 (GraphPad), one‐way analysis of variance (ANOVA), and Dunnett's test to assess differences in pharmacological parameters among groups. *p* < 0.05. The metagenomic data were subjected to permutational multivariate ANOVA to evaluate the significance of group differences via principal component analysis and PCoA. The Wilcoxon nonparametric test was applied for significant differences among the groups. For multiple tests, adjusted *p* values were calculated using Benjamini–Hochberg's correction. *p* < 0.05.

## AUTHOR CONTRIBUTIONS

You‐Gui Li and Chong‐Ming Wu conceived and designed the study. Shi Zhong and Yu‐Qing Sun performed the experiments with the help of Jin‐Xi Huo, Wen‐Yi Xu, Ya‐Nan Yang, and Wei‐Jie Wu. You‐Gui Li, Chong‐Ming Wu, Yong‐Xin Liu, and Jun‐Bo Yang analyzed the data. Wen‐Yi Xu, Ya‐Nan Yang, You‐Gui Li, and Chong‐Ming Wu prepared the manuscript. All the authors reviewed and approved the final manuscript.

## CONFLICT OF INTEREST STATEMENT

Wen‐Yi Xu is an employee of Beijing QuantiHealth Technology Co., Ltd. The other authors declare no conflict of interest in this work.

## ETHICS STATEMENT

Animal experiments were conducted with the approval of the Institutional Animal Care and Use Committee (IACUC) of the Zhejiang Academy of Agricultural Sciences under protocol number 2021ZAASLA30. All procedures adhered to the Guidelines for the Care and Use of Laboratory Animals issued by the Chinese Council on Animal Research.

## Supporting information

The online version contains supplementary figures and tables available.

**Figure S1.** Chemical profile of the SH extract by HPLC‐TOF/MS analysis.
**Figure S2.** The anticolitic effect of SH in DSS‐induced mice.
**Figure S3.** The anti‐inflammatory effect of SH in DSS‐induced mice.
**Figure S4.** The protective effect of SH on the intestinal mucosal barrier in DSS‐induced mice.
**Figure S5.** The effect of DSS‐modulated changes in the gut microbiota on colitis symptoms in DSS‐induced mice.
**Figure S6.** The protective effect of the SH‐modulated gut microbiota on colonic tissue in DSS‐induced mice.
**Figure S7.** *Alistipes* is a SH‐enriched genus involved in the colitis‐ameliorating effect of SH.
**Figure S8.** S3 One strain of *Alistipes onderdonkii* barely exerts its anticolitis effect.
**Figure S9.** 5‐Hydroxyindole‐3‐acetic acid (5HIAA) is an SH‐enriched gut microbial metabolite that mediates the anticolitis effect of SH.
**Figure S10.** Treatment with 5HIAA alleviates DSS‐induced inflammation and intestinal mucosal barrier injury in C57BL/6 mice.
**Figure S11.** Treatment with 5HIAA activates the AhR signaling pathway in Caco2 cells.
**Table S1.** Characterization of the chemical constituents of SH by HPLC‐TOF/MS.
**Table S2.** qRT‒PCR primers used in this work.

 

## Data Availability

The data are available upon reasonable request. All the relevant data were included in the article or uploaded as supplemental online materials. The 16S rRNA gene sequencing data were deposited in the NCBI database (SRA Bioproject No. PRJNA821597, https://www.ncbi.nlm.nih.gov/bioproject/PRJNA821597/). The metagenomic and assembly related data have been submitted to the Genome Sequence Archive under the project identifier PRJCA021089, with accession numbers CRA013411 for metagenomic sequencing and CRA013410 for bacterial genome sequencing at the National Genomics Data Center, China National Center for Bioinformation/Beijing Institute of Genomics, Chinese Academy of Sciences and are publicly available at https://ngdc.cncb.ac.cn/gsa. The data and scripts for metagenomic analysis are saved in GitHub https://github.com/YongxinLiu/EasyMetagenome. The data and scripts for visualization are saved in GitHub https://github.com/joybio/graphlan_plot. Supplementary materials (figures, tables, scripts, graphical abstract, slides, videos, Chinese translated version and update materials) may be found in the online DOI or iMeta Science http://www.imeta.science/.

## References

[1] Kaplan, Gilaad G. 2015. “The Global Burden of IBD: From 2015 to 2025.” Nature Reviews Gastroenterology & Hepatology 12: 720–727. 10.1038/nrgastro.2015.150 26323879

[2] Kobayashi, Taku , Britta Siegmund , Catherine Le Berre , Shu Chen Wei , Marc Ferrante , Bo Shen , Charles N. Bernstein , et al. 2020. “Ulcerative Colitis.” Nature Reviews Disease Primers 6: 74. 10.1038/s41572-020-0205-x 32913180

[3] Roda, Giulia , Siew Chien Ng , Paulo Gustavo Kotze , Marjorie Argollo , Remo Panaccione , Antonino Spinelli , Arthur Kaser , Laurent Peyrin‐Biroulet , and Silvio Danese . 2020. “Crohn's Disease.” Nature Reviews Disease Primers 6: 22. 10.1038/s41572-020-0156-2 32242028

[4] Roda, Giulia , Bindia Jharap , Narula Neeraj , and Jean‐Frederic Colombel . 2016. “Loss of Response to Anti‐TNFs: Definition, Epidemiology, and Management.” Clinical and Translational Gastroenterology 7: e135. 10.1038/ctg.2015.63 26741065 PMC4737871

[5] Ni, Josephine , Gary D. Wu , Lindsey Albenberg , and Vesselin T. Tomov . 2017. “Gut Microbiota and IBD: Causation or Correlation?” Nature Reviews Gastroenterology & Hepatology 14: 573–584. 10.1038/nrgastro.2017.88 28743984 PMC5880536

[6] Gao, Yunyun , Danyi Li , and Yongxin Liu . 2023. “Microbiome Research Outlook: Past, Present, and Future.” Protein & Cell 14: 709–712. 10.1093/procel/pwad031 37219087 PMC10599639

[7] Machiels, Kathleen , Marie Joossens , João Sabino , Vicky De Preter , Ingrid Arijs , Venessa Eeckhaut , Vera Ballet , et al. 2014. “A Decrease of the Butyrate‐Producing Species *Roseburia hominis* and *Faecalibacterium prausnitzii* Defines Dysbiosis in Patients with Ulcerative Colitis.” Gut 63: 1275–1283. 10.1136/gutjnl-2013-304833 24021287

[8] Vieira, Erica L. M. , Alda J. Leonel , Alexandre P. Sad , Nathália R. M. Beltrão , Thaís F. Costa , Talita M. R. Ferreira , Ana C. Gomes‐Santos , et al. 2012. “Oral Administration of Sodium Butyrate Attenuates Inflammation and Mucosal Lesion in Experimental Acute Ulcerative Colitis.” The Journal of Nutritional Biochemistry 23: 430–436. 10.1016/j.jnutbio.2011.01.007 21658926

[9] Lavelle, Aonghus , and Harry Sokol . 2020. “Gut Microbiota‐Derived Metabolites as Key Actors in Inflammatory Bowel Disease.” Nature Reviews Gastroenterology & Hepatology 17: 223–237. 10.1038/s41575-019-0258-z 32076145

[10] Aoki, Reiji , Ayako Aoki‐Yoshida , Chise Suzuki , and Yoshiharu Takayama . 2018. “Indole‐3‐Pyruvic Acid, an Aryl Hydrocarbon Receptor Activator, Suppresses Experimental Colitis in Mice.” The Journal of Immunology 201: 3683–3693. 10.4049/jimmunol.1701734 30429284

[11] Busbee, Philip B. , Lorenzo Menzel , Haider Alrafas , Nicholas Dopkins , William Becker , Kathryn Miranda , Chaunbing Tang , et al. 2020. “Indole‐3‐Carbinol Prevents Colitis and Associated Microbial Dysbiosis In an IL‐22–Dependent Manner.” JCI Insight 5: e127551. 10.1172/jci.insight.127551 31941837 PMC7030851

[12] Lamas, Bruno , Mathias L. Richard , Valentin Leducq , Hang‐Phuong Pham , Marie‐Laure Michel , Gregory Da Costa , Chantal Bridonneau , et al. 2016. “CARD9 Impacts Colitis by Altering Gut Microbiota Metabolism of Tryptophan into Aryl Hydrocarbon Receptor Ligands.” Nature Medicine 22: 598–605. 10.1038/nm.4102 PMC5087285 27158904

[13] Peng, Chunting , Chensi Wu , Xiaolan Xu , Liya Pan , Zhuoqi Lou , Yanhong Zhao , Haiyin Jiang , Zebao He , and Bing Ruan . 2021. “Indole‐3‐Carbinol Ameliorates Necroptosis and Inflammation of Intestinal Epithelial Cells in Mice with Ulcerative Colitis by Activating Aryl Hydrocarbon Receptor.” Experimental Cell Research 404: 112638. 10.1016/j.yexcr.2021.112638 34015312

[14] Yang, Wuqi , Daoyuan Ren , Yan Zhao , Lei Liu , and Xingbin Yang . 2021. “Fuzhuan Brick Tea Polysaccharide Improved Ulcerative Colitis in Association with Gut Microbiota‐Derived Tryptophan Metabolism.” Journal of Agricultural and Food Chemistry 69: 8448–8459. 10.1021/acs.jafc.1c02774 34313113

[15] Hao, Panpan , Fan Jiang , Jing Cheng , Lianyue Ma , Yun Zhang , and Yuxia Zhao . 2017. “Traditional Chinese Medicine for Cardiovascular Disease.” Journal of the American College of Cardiology 69: 2952–2966. 10.1016/j.jacc.2017.04.041 28619197

[16] Atanasov, Atanas G. , Sergey B. Zotchev , Verena M. Dirsch , and Claudiu T. Supuran . 2021. “Natural Products in Drug Discovery: Advances and Opportunities.” Nature Reviews Drug Discovery 20: 200–216. 10.1038/s41573-020-00114-z 33510482 PMC7841765

[17] Sun, Zhongmei , Junxiang Li , Yi Dai , Wenting Wang , Rui Shi , Zhibin Wang , Panghua Ding , et al. 2020. “Indigo Naturalis Alleviates Dextran Sulfate Sodium‐Induced Colitis in Rats via Altering Gut Microbiota.” Frontiers in Microbiology 11: 731. 10.3389/fmicb.2020.00731 32425906 PMC7203728

[18] Wu, Chongming , Yu Tian , Jiaqi Yu , Rong Zhang , Xiaopo Zhang , and Peng Guo . 2019. “The *Pandanus tectorius* Fruit Extract (PTF) Modulates the Gut Microbiota and Exerts Anti‐Hyperlipidaemic Effects.” Phytomedicine 58: 152863. 10.1016/j.phymed.2019.152863 30836215

[19] Wu, Minna , Puze Li , Yunying An , Jie Ren , Dong Yan , Jiazeng Cui , Duan Li , et al. 2019. “Phloretin Ameliorates Dextran Sulfate Sodium‐Induced Ulcerative Colitis in Mice by Regulating the Gut Microbiota.” Pharmacological Research 150: 104489. 10.1016/j.phrs.2019.104489 31689519

[20] Jiang, Wen‐Ping , Jeng‐Shyan Deng , Shyh‐Shyun Huang , Sheng‐Hua Wu , Chin‐Chu Chen , Jung‐Chun Liao , Hung‐Yi Chen , Hui‐Yi Lin , and Guan‐Jhong Huang . 2021. “ *Sanghuangporus sanghuang* Mycelium Prevents Paracetamol‐Induced Hepatotoxicity Through Regulating the MAPK/NF‐κB, Keap1/Nrf2/HO‐1, TLR4/PI3K/Akt, and CaMKKβ/LKB1/AMPK Pathways and Suppressing Oxidative Stress and Inflammation.” Antioxidants 10: 897. 10.3390/antiox10060897 34199606 PMC8226512

[21] Lin, Wangching , Jeng‐Shyan Deng , Shyh‐Shyun Huang , Shenghua Wu , Chin‐Chu Chen , Wanrong Lin , Huiyi Lin , and Guan‐Jhong Huang . 2017. “Anti‐Inflammatory Activity of *Sanghuangporus sanghuang* Mycelium.” International Journal of Molecular Sciences 18: 347. 10.3390/ijms18020347 28178212 PMC5343882

[22] Qu, Yidi , Hongxin Yang , Siyu Li , Lanzhou Li , Yu Li , and Di Wang . 2023. “The Involvement of Th1 Cell Differentiation in the Anti‐Tumor Effect of Purified Polysaccharide From *Sanghuangporus vaninii* In Colorectal Cancer Via Multi‐Omics Analysis.” International Journal of Biological Macromolecules 237: 123927. 10.1016/j.ijbiomac.2023.123927 36889619

[23] Qiu, Ping , Jingqun Liu , Lisha Zhao , Pinghu Zhang , Weike Wang , Dan Shou , Jinjun Ji , et al. 2022. “Inoscavin A, a Pyrone Compound Isolated from a *Sanghuangporus vaninii* Extract, Inhibits Colon Cancer Cell Growth and Induces Cell Apoptosis Via the Hedgehog Signaling Pathway.” Phytomedicine 96: 153852. 10.1016/j.phymed.2021.153852 35026508

[24] Liu, Jinze , Jinyue Song , WeiJia Chen , Li Sun , Yan Zhao , Ying Zong , Zhongmei He , and Rui Du . 2024. “Assessment of Cytotoxicity, Acute, Subacute Toxicities and Antioxidant Activities (*In Vitro*) of *Sanghuangporus vaninii* Crude Polysaccharide.” Journal of Ethnopharmacology 319: 117284. 10.1016/j.jep.2023.117284 37844741

[25] Wang, Hao , Jinxin Ma , Dongmei Wu , Neng Gao , Jing Si , and Baokai Cui . 2023. “Identifying Bioactive Ingredients and Antioxidant Activities of Wild *Sanghuangporus* Species of Medicinal Fungi.” Journal of Fungi 9: 242. 10.3390/jof9020242 36836356 PMC9959451

[26] Wu, Di , Xuemei Yuan , Ruijie Zhou , Wanchao Chen , Wen Li , Zhengpeng Li , Xueyin Li , et al. 2023. “Aqueous Extract of *Sanghuangporus baumii* Induces Autophagy to Inhibit Cervical Carcinoma Growth.” Food & Function 14: 2374–2384. 10.1039/d2fo02887e 36779533

[27] Huang, Zirui , Yun Liu , Xiaoyan Liu , Kewen Chen , Wenyu Xiong , Yuyang Qiu , Xiaoyu He , Bin Liu , and Feng Zeng . 2022. “ *Sanghuangporus vaninii* Mixture Ameliorated Type 2 Diabetes Mellitus and Altered Intestinal Microbiota in Mice.” Food & Function 13: 11758–11769. 10.1039/d2fo02268k 36285656

[28] Teng, Yun , Yi Ren , Mohammed Sayed , Xin Hu , Chao Lei , Anil Kumar , Elizabeth Hutchins , et al. 2018. “Plant‐Derived Exosomal microRNAs Shape the Gut Microbiota.” Cell Host & Microbe 24: 637–652.e638. 10.1016/j.chom.2018.10.001 30449315 PMC6746408

[29] Peyrin‐Biroulet, Laurent , Silvio Danese , Marjorie Argollo , Lieven Pouillon , Spyros Peppas , Marien Gonzalez‐Lorenzo , Theodore Lytras , and Stefanos Bonovas . 2019. “Loss of Response to Vedolizumab and Ability of Dose Intensification to Restore Response in Patients with Crohn's Disease or Ulcerative Colitis: A Systematic Review and Meta‐Analysis.” Clinical Gastroenterology and Hepatology 17: 838–846.e2. 10.1016/j.cgh.2018.06.026 29935327

[30] Ananthakrishnan, Ashwin N. , Chengwei Luo , Vijay Yajnik , Hamed Khalili , John J. Garber , Betsy W. Stevens , Thomas Cleland , and Ramnik J. Xavier . 2017. “Gut Microbiome Function Predicts Response to Anti‐Integrin Biologic Therapy in Inflammatory Bowel Diseases.” Cell Host & Microbe 21: 603–610.e603. 10.1016/j.chom.2017.04.010 28494241 PMC5705050

[31] Sun, Yuqing , Jinxi Huo , Shi Zhong , Jianxun Zhu , Yougui Li , and Xiaojun Li . 2021. “Chemical Structure and Anti‐Inflammatory Activity of a Branched Polysaccharide Isolated From *Phellinus baumii* .” Carbohydrate Polymers 268: 118214. 10.1016/j.carbpol.2021.118214 34127216

[32] Zuo, Kang , Kaijing Tang , Yue Liang , Yifan Xu , Kangliang Sheng , Xiaowei Kong , Jingmin Wang , et al. 2020. “Purification and Antioxidant and Anti‐Inflammatory Activity of Extracellular Polysaccharopeptide from Sanghuang Mushroom, *Sanghuangporus lonicericola* .” Journal of the Science of Food and Agriculture 101: 1009–1020. 10.1002/jsfa.10709 32767366

[33] Huo, Jinxi , Shi Zhong , Xin Du , Yinglong Cao , Wenqiong Wang , Yuqing Sun , Yu Tian , et al. 2020. “Whole‐Genome Sequence of *Phellinus gilvus* (Mulberry Sanghuang) Reveals Its Unique Medicinal Values.” Journal of Advanced Research 24: 325–335. 10.1016/j.jare.2020.04.011 32455007 PMC7235939

[34] Sun, Yuqing , Shi Zhong , Bo Deng , Qinsheng Jin , Jie Wu , Jinxi Huo , Jianxun Zhu , Cheng Zhang , and Yougui Li . 2020. “Impact of *Phellinus gilvus* Mycelia on Growth, Immunity and Fecal Microbiota in Weaned Piglets.” PeerJ 8: e9067. 10.7717/peerj.9067 32377455 PMC7194088

[35] Knights, Dan , Kara G. Lassen , and Ramnik J. Xavier . 2013. “Advances in Inflammatory Bowel Disease Pathogenesis: Linking Host Genetics and the Microbiome.” Gut 62: 1505–1510. 10.1136/gutjnl-2012-303954 24037875 PMC3822528

[36] Rossen, Noortje G. , Susana Fuentes , Mirjam J. van der Spek , Jan G. Tijssen , Jorn H. A. Hartman , Ann Duflou , Mark Löwenberg , et al. 2015. “Findings from a Randomized Controlled Trial of Fecal Transplantation for Patients with Ulcerative Colitis.” Gastroenterology 149: 110–118.e4. 10.1053/j.gastro.2015.03.045 25836986

[37] Hayashi, Atsushi , Toshiro Sato , Nobuhiko Kamada , Yohei Mikami , Katsuyoshi Matsuoka , Tadakazu Hisamatsu , Toshifumi Hibi , et al. 2013. “A Single Strain of *Clostridium butyricum* Induces Intestinal IL‐10‐Producing Macrophages to Suppress Acute Experimental Colitis in Mice.” Cell Host & Microbe 13: 711–722. 10.1016/j.chom.2013.05.013 23768495

[38] Qiu, Xinyun , Feng Zhang , Xi Yang , Na Wu , Weiwei Jiang , Xia Li , Xiaoxue Li , and Yulan Liu . 2015. “Changes in the Composition of Intestinal Fungi and Their Role in Mice with Dextran Sulfate Sodium‐Induced Colitis.” Scientific Reports 5: 10416. 10.1038/srep10416 26013555 PMC4445066

[39] Moschen, Alexander R. , Romana R. Gerner , Jun Wang , Victoria Klepsch , Timon E. Adolph , Simon J. Reider , Hubert Hackl , et al. 2016. “Lipocalin 2 Protects From Inflammation and Tumorigenesis Associated with Gut Microbiota Alterations.” Cell Host & Microbe 19: 455–469. 10.1016/j.chom.2016.03.007 27078067

[40] Dziarski, Roman , Shin Yong Park , Des Raj Kashyap , Scot E. Dowd , and Dipika Gupta . 2016. “Pglyrp‐Regulated Gut Microflora *Prevotella falsenii*, *Parabacteroides distasonis* and *Bacteroides eggerthii* Enhance and *Alistipes finegoldii* Attenuates Colitis in Mice.” PloS one 11: e0146162. 10.1371/journal.pone.0146162 26727498 PMC4699708

[41] Patnode, Michael L. , Janaki L. Guruge , Juan J. Castillo , Garret A. Couture , Vincent Lombard , Nicolas Terrapon , Bernard Henrissat , Carlito B. Lebrilla , and Jeffrey I. Gordon . 2021. “Strain‐Level Functional Variation in the Human Gut Microbiota Based on Bacterial Binding to Artificial Food Particles.” Cell Host & Microbe 29: 664–673.e665. 10.1016/j.chom.2021.01.007 33571448 PMC8529970

[42] Zhai, Rui , Xinhe Xue , Liying Zhang , Xin Yang , Liping Zhao , and Chenhong Zhang . 2019. “Strain‐Specific Anti‐Inflammatory Properties of Two *Akkermansia muciniphila* Strains on Chronic Colitis in Mice.” Frontiers in Cellular and Infection Microbiology 9: 239. 10.3389/fcimb.2019.00239 31334133 PMC6624636

[43] Sugimoto, Shinya , Makoto Naganuma , and Takanori Kanai . 2016. “Indole Compounds May Be Promising Medicines for Ulcerative Colitis.” Journal of Gastroenterology 51: 853–861. 10.1007/s00535-016-1220-2 27160749

[44] Agus, Allison , Julien Planchais , and Harry Sokol . 2018. “Gut Microbiota Regulation of Tryptophan Metabolism in Health and Disease.” Cell Host & Microbe 23: 716–724. 10.1016/j.chom.2018.05.003 29902437

[45] Gutiérrez‐Vázquez, Cristina , and Francisco J. Quintana . 2018. “Regulation of the Immune Response by the Aryl Hydrocarbon Receptor.” Immunity 48: 19–33. 10.1016/j.immuni.2017.12.012 29343438 PMC5777317

[46] Stockinger, Brigitta , Kathleen Shah , and Emma Wincent . 2021. “AHR in the Intestinal Microenvironment: Safeguarding Barrier Function.” Nature Reviews Gastroenterology & Hepatology 18: 559–570. 10.1038/s41575-021-00430-8 33742166 PMC7611426

[47] Shima, Hisato , Kensuke Sasaki , Takehiro Suzuki , Chikahisa Mukawa , Ten Obara , Yuki Oba , Akihiro Matsuo , et al. 2017. “A Novel Indole Compound MA‐35 Attenuates Renal Fibrosis by Inhibiting Both TNF‐α and TGF‐β1 Pathways.” Scientific Reports 7: 1884. 10.1038/s41598-017-01702-7 28507324 PMC5432497

[48] Alexeev, Erica E. , Jordi M. Lanis , Daniel J. Kao , Eric L. Campbell , Caleb J. Kelly , Kayla D. Battista , Mark E. Gerich , et al. 2018. “Microbiota‐Derived Indole Metabolites Promote Human and Murine Intestinal Homeostasis Through Regulation of Interleukin‐10 Receptor.” The American Journal of Pathology 188: 1183–1194. 10.1016/j.ajpath.2018.01.011 29454749 PMC5906738

[49] Sun, Yuqing , Shi Zhong , Jiaqi Yu , Jianxun Zhu , Dongfeng Ji , Guiyan Hu , Chongming Wu , and Yougui Li . 2018. “The Aqueous Extract of *Phellinus igniarius* (SH) Ameliorates Dextran Sodium Sulfate‐Induced Colitis in C57BL/6 Mice.” PloS one 13: e0205007. 10.1371/journal.pone.0205007 30289941 PMC6173430

[50] Panés, Julián , Montserrat Aceituno , Fèlix Gil , Rosa Miquel , Josep M. Piqué , Azucena Salas , and Peter McLean . 2007. “Efficacy of an Inhibitor of Adhesion Molecule Expression (GI270384X) in the Treatment of Experimental Colitis.” American Journal of Physiology—Gastrointestinal and Liver Physiology 293: G739–G748. 10.1152/ajpgi.00059.2007 17656448

[51] Thomaz, Ana C. , Vishakh Iyer , Taylor J. Woodward , and Andrea G. Hohmann . 2021. “Fecal Microbiota Transplantation and Antibiotic Treatment Attenuate Naloxone‐Precipitated Opioid Withdrawal in Morphine‐Dependent Mice.” Experimental Neurology 343: 113787. 10.1016/j.expneurol.2021.113787 34153321 PMC8477666

[52] Wen, Tao , Guoqing Niu , Tong Chen , Qirong Shen , Jun Yuan , and Yongxin Liu . 2023. “The Best Practice for Microbiome Analysis Using R.” Protein & Cell 14: 713–725. 10.1093/procel/pwad024 37128855 PMC10599642

[53] Liu, Yong‐Xin , Lei Chen , Tengfei Ma , Xiaofang Li , Maosheng Zheng , Xin Zhou , Liang Chen , et al. 2023. “EasyAmplicon: An Easy‐to‐Use, Open‐Source, Reproducible, and Community‐Based Pipeline for Amplicon Data Analysis in Microbiome Research.” iMeta 2: e83. 10.1002/imt2.83 38868346 PMC10989771

[54] Chen, Tong , Yong‐Xin Liu , and Luqi Huang . 2022. “ImageGP: An Easy‐to‐Use Data Visualization Web Server for Scientific Researchers.” iMeta 1: e5. 10.1002/imt2.5 38867732 PMC10989750

[55] Peng, Kai , Yongxin Liu , Xinran Sun , Qiaojun Wang , Pengcheng Du , Yunzeng Zhang , Mianzhi Wang , Zhiqiang Wang , and Ruichao Li . 2023. “Long‐Read Metagenomic Sequencing Reveals That High‐Copy Small Plasmids Shape the Highly Prevalent Antibiotic Resistance Genes in Animal Fecal Microbiome.” Science of the Total Environment 893: 164585. 10.1016/j.scitotenv.2023.164585 37269991

[56] Chen, Shifu . 2023. “Ultrafast One‐Pass FASTQ Data Preprocessing, Quality Control, and Deduplication Using Fastp.” iMeta 2: e107. 10.1002/imt2.107 38868435

[57] De Coster, Wouter , Svenn D'Hert , Darrin T. Schultz , Marc Cruts , and Christine Van Broeckhoven . 2018. “NanoPack: Visualizing and Processing Long‐Read Sequencing Data.” Bioinformatics 34: 2666–2669. 10.1093/bioinformatics/bty149 29547981 PMC6061794

[58] Phillippy, Adam M. , Ryan R. Wick , Louise M. Judd , Claire L. Gorrie , and Kathryn E. Holt . 2017. “Unicycler: Resolving Bacterial Genome Assemblies from Short and Long Sequencing Reads.” PLoS Computational Biology 13: e1005595. 10.1371/journal.pcbi.1005595 28594827 PMC5481147

[59] Grant, Jason R. , Eric Enns , Eric Marinier , Arnab Mandal , Emily K. Herman , Chih‐yu Chen , Morag Graham , Gary Van Domselaar , and Paul Stothard . 2023. “Proksee: In‐Depth Characterization and Visualization of Bacterial Genomes.” Nucleic Acids Research 51: W484–W492. 10.1093/nar/gkad326 37140037 PMC10320063

