# Supplementary material for: The gut microbiota‐aromatic hydrocarbon receptor (AhR) axis mediates the anticolitic effect of polyphenol‐rich extracts from Sanghuangporus
Source: Imeta. 2024 Mar 11;3(2):e180. doi: 10.1002/imt2.180 (PMC11170970; doi:10.1002/imt2.180)

# Supporting information to

# The gut microbiota-aromatic hydrocarbon receptor (AhR) axis mediates the anti-colitis effect of polyphenol-rich extract from *Sanghuangporus*

**Running title:** *Sanghuangporus* polyphenol extract alleviates colitis via gut microbes-AhR axis

Shi Zhong^1#^, Yu-Qing Sun^1#^, Jin-Xi Huo^1^, Wen-Yi Xu^2^, Ya-Nan Yang^3^, Jun-Bo Yang^4^, Wei-Jie Wu^1^, Yong-Xin Liu^4*^, Chong-Ming Wu^3*^, You-Gui Li^1*^

^1^Zhejiang Academy of Agricultural Sciences, Hangzhou 310021, China

^2^Beijing QuantiHealth Technology Co., Ltd., Beijing 100070, China

^3^School of Chinese Materia Medica, Tianjin University of Traditional Chinese Medicine, Tianjin 301617, China

^4^Shenzhen Branch, Guangdong Laboratory of Lingnan Modern Agriculture, Genome Analysis Laboratory of the Ministry of Agriculture and Rural Affairs, Agricultural Genomics Institute at Shenzhen, Chinese Academy of Agricultural Sciences, Shenzhen, Guangdong 518120, China

^#^These authors contributed equally to this work

^*^Correspondence: <liyougui3@126.com> (You-Gui Li); [chomingwu@163.com](file:///D:\\桑黄\\桑黄抗炎症实验\\lyg-DSS肠炎\\AHR信号通路补充实验\\测试数据\\manucript\\imeta%20桑黄重新作图2023-8-15\\iMeta投稿\\iMeta%20投稿\\revised%201\\revised%201\\iMeta-R1上传稿\\imeta-R2\\imeta-R2-上传稿\\chomingwu@163.com) (Chong-Ming Wu); [liuyongxin@caas.cn](file:///D:\\桑黄\\桑黄抗炎症实验\\lyg-DSS肠炎\\AHR信号通路补充实验\\测试数据\\manucript\\imeta%20桑黄重新作图2023-8-15\\iMeta投稿\\iMeta%20投稿\\revised%201\\revised%201\\iMeta-R1上传稿\\imeta-R2\\imeta-R2-上传稿\\liuyongxin@caas.cn) (Yong-Xin Liu)

# SUPPLEMENTAL MATERIALS

**Materials and Methods**

Figure S1 Chemical profile of the SH extract by HPLC-TOF/MS analysis.

Figure S2 The anticolitic effect of SH in DSS-induced mice.

Figure S3 The anti-inflammatory effect of SH in DSS-induced mice.

Figure S4 The protective effect of SH on the intestinal mucosal barrier in DSS-induced mice.

Figure S5 The effect of DSS-modulated changes in the gut microbiota on colitis symptoms in DSS-induced mice.

Figure S6 The protective effect of the SH-modulated gut microbiota on colonic tissue in DSS-induced mice.

Figure S7 *Alistipes* is a SH-enriched genus involved in the colitis-ameliorating effect of SH.

Figure S8 S3 One strain of *Alistipes onderdonkii* barely exerts its anti-colitis effect.

Figure S9 5-Hydroxyindole-3-acetic acid (5HIAA) is an SH-enriched gut microbial metabolite that mediates the anti-colitis effect of SH.

Figure S10 Treatment with 5HIAA alleviates DSS-induced inflammation and intestinal mucosal barrier injury in C57BL/6 mice.

Figure S11 Treatment with 5HIAA activates the AhR signaling pathway in Caco2 cells.

Table S1 Characterization of the chemical constituents of SH by HPLC-TOF/MS.

Table S2 qRT‒PCR primers used in this work.

**Materials and Methods**

**SH preparation and chemical analysis**

The preparation of SH followed a previously described method with some modification [1]. Fruiting bodies of cultured *Sanghuangporus* were sourced from the Sericultural Research Institute, Zhejiang Academy of Agricultural Science (Hangzhou, China). SH powder (approximately 500 g) was suspended in 60% ethanol (1:60, w/v). Following 30 min of ultrasound treatment, reflux extraction was conducted at 90 ℃ for 1 h. The resulting extract underwent vacuum filtration, and the filtrate was collected and centrifuged (10,000 rpm, 10 min). The solvent from the supernatant was evaporated at 60 ℃ in a rotary evaporator under low pressure, followed by lyophilization to dryness at -50 ℃ under low pressure. The crude extract was dissolved in distilled water (5mg/mL) and purified using an AB-8 macroporous adsorption resin column (Solarbio, Beijing, China) with sequential elution using distilled water, 30% ethanol and 70% ethanol. The eluent with 70% ethanol was collected, concentrated, and lyophilized to obtain the purified SH. The total polyphenol content of SH was determined to be 93.86% ±2.78% using the Folin-Ciocalteu, as previously reported [2]. The obtained SH powder was stored at 4 ℃ for subsequent experiments.

The chemical compounds of the SH were analyzed using high-performance liquid chromatography/time-of-flight mass spectrometry (HPLC-TOF/MS) on an Agilent 1260 Infinity II UHPLC system coupled to an Abscix Triple TOF/MS instrument. Chromatographic separation utilized an Exsil Plus EPS C18 column (5 μm 4.6 × 250 mm, Agilent, USA). Mobile phase A consisted of water containing 0.1% formic acid, while mobile phase B comprised acetonitrile. The column temperature was maintained at 30 ℃, and the HPLC flow rate was set at 1 mL/min. A mobile phase gradient was employed, with the percentage of B in A changing as follows: initial concentration, 10% B; 10 min; 25% B; 30 min, 35% B; 50 min, 55% B; 60 min, 90% B. The eluted fraction of each peak was collected and subjected to analysis by high-resolution electrospray ionization mass spectrometry (HRESIMS) using an Agilent G6224A TOF spectrometer. Mass spectrometry experiments were conducted on a TOF mass spectrometer with an electrospray ionization (ESI) interface (Agilent, USA). Positive ESI conditions were set as follows: gas temperature, 350 ℃; drying gas, 9 L/min, nebulizer, 40 psi, capillary, 3500.

**Animal experiments**

Animal experiments were conducted with approval from the Institutional Animal Care and Use Committee (IACUC) of the Zhejiang Academy of Agricultural Sciences, under protocol number 2021ZAASLA30. All procedures adhered to the Guidelines for the Care and Use of Laboratory Animals issued by the Chinese Council on Animal Research. For all experiments, male C57BL/6 mice (6-week-old) were procured from Shanghai Experimental Animal Center (China) and housed under controlled temperature and humidity conditions with a 12h/12h light/dark cycle. They received normal rodent chow and sterile water.

To assess the anti-colitis effect of *A. onderdonkii*, three human feces-derived *A. onderdonkii* strains were purchased from Beijing QuantiHealth Technology Co., Ltd. (Beijing, China). Male C57BL/6 mice were randomly into four groups with 8 animals in each group, i.e., Vehicle, *A. onderdonkii*-1 (FDB8), *A. onderdonkii*-2 (FDFM), and *A. onderdonkii*-3 (FDPA). The Vehicle group was given an equal volume of the YCFA bacterial culture medium, while the other three groups were orally gavaged with respective *A. onderdonkii* strain (10^9^ CFU/animal per day). After treatment for 4 days, all animals were simultaneously given DSS in the drinking water (3.0% w/v) for additional seven days. Body weight and DAI were assessed every day. At the end of the experiment, mice were fasted overnight, blood samples were collected for analysis and the colon tissues were resected and frozen at -80 ℃ for biochemical measurements.

To assess the anti-colitis effect of 5HIAA and IAA, C57BL/6 mice were randomly into four groups with 8 animals in each group, i.e., Normal control (NC), DSS, DSS+IAA and DSS+5HIAA groups. NC group was given an equal volume of the distilled water, while the other 3 groups were first established chronic colitis with DSS supplementation in the drinking water (1.0% w/v), the DSS+5HIAA and DSS+IAA groups were simultaneously treated with 5HIAA (5 mg/kg) and IAA (5 mg/kg), respectively. Body weight was measured every 3 days, and DAI was evaluated on Day 42. At the end of the experiment, mice were fasted overnight, blood samples were collected for analysis and the colon tissues were resected and fixed in 4% paraformaldehyde for histological analysis or frozen at -80 ℃ for biochemical measurements.

To assess the role of AhR in the anti-colitis effect of SH, C57BL/6 mice were randomly divided into 5 groups with 8 animals in each group, including NC, DSS, DSS+AhRi, DSS+AhRi+SH, DSS+AhRi+5HIAA groups. The NC group was given an equal volume of the distilled water, the other groups were established acute colitis with DSS supplementation in the drinking water (3.0% w/v). The doses of SH and 5HIAA were 400 mg/kg and 5 mg/kg, respectively, while AhRi groups were simultaneously treated with StemRegenin 1 (50 mg/kg) by gavage. During the experimental period, body weight was measured every day, DAI was evaluated on Day 10. At the end of the experiment, mice were fasted overnight, blood samples were collected for analysis and the colon tissues were resected and fixed in 4% paraformaldehyde for histological analysis or frozen at -80 ℃ for biochemical measurements.

**Histology and immunofluorescence**

Colon length of each mouse was measured and recorded after tissue resections and a segment of the colon tissues from each mouse was fixed in 4% paraformaldehyde, embedded in paraffin, and sectioned into 5 μm-thick sections. Multiple sections from consecutive sections were stained with hematoxylin and eosin (H&E) or Alcian blue for histology assessment under a light microscopy (Nikon, Japan).

For immunofluorescence, the rehydrated tissue sections were blocked in 5% bovine serum albumin (BSA) for 1 h and incubated overnight at 4 ℃ with primary antibody, rabbit anti-Claudin-4 antibody (16195-1-AP; 1:500; Proteintech). The sections were then washed with PBS and incubated for 1 h at room temperature with Alexa Fluor 488-conjugated donkey anti-rabbit IgG (ab150073, 1:250; Abcam). DAPI staining solution (C1002, Beyotime) was used to stain cell nuclei. The immunostained sections were reviewed and scored under a florescent microscope (Nikon, Japan).

**Quantitative real-time PCR (qRT-PCR)**

Total cellular RNA was isolated from mouse colonic tissues or primary macrophages using TaKaRa MiniBEST universal RNA extraction kit (TaKaRa, Japan) and reversely transcribed into cDNA using PrimeScriptTM RT master mix (TaKaRa, Japan) according to the manufacturers’ instructions. PCR reactions were performed using SYBR ®Premix Ex Taq™ (TaKaRa, Japan) in a StepOnePlus real-time PCR system (Applied Biosystems). β-actin was used as an internal control. All primers sequences used in the study were listed in **Table S2**.

**Western blot**

Homogenized tissues were lysed in RIPA buffer (R0020, Solarbio) containing protease inhibitor cocktails (B14001; Bimake). Samples were resolved in SDS-PAGE gels and then transferred onto PVDF membranes. After blocking in 5% non-fat milk, membranes were incubated with indicated primary antibodies and secondary antibodies following the regular procedures. Protein signals were visualized by the chemiluminescence imaging system (Amersham Imager 600, GE Healthcare Life Sciences). Primary antibodies used in the study included antibodies against Occludin (13409-1-AP,1:1000; Proteintech), Claudin-3 (16456-1-AP, 1:1000; Proteintech), Claudin-4 ([16195-1-AP](https://www.ptgcn.com/products/CLDN4-specific-Antibody-16195-1-AP.htm),1:1000; Proteintech), and β-actin (60008-1-Ig,1:5000; Proteintech).

**Metabolomic analysis**

Metabolomic analysis of fecal materials was performed using the metabolomic platform of Guangzhou Genedenovo Biotechnology Co., Ltd., following established protocols [3]. Briefly, approximately 100 mg of colonic contents were resuspended with 500 μL of 80% methanol (prechilled at 4 ℃) through vigorous vortexing. The suspension was incubated on ice for 5 min and then centrifuged at 15,000 × g for 20 min at 4 ℃. The supernatant was transferred into a new tube and diluted with distilled water to achieve a final methanol concentration of 53%. After a further centrifugation at 15,000 × g for 20 min at 4 ℃, the supernatant was collected for testing by the ultra-high performance liquid chromatography-tandem mass spectrometry (UHPLC-MS/MS) system.

Chromatographic separation was performed on an Agilent 1200 Series HPLC instrument (Agilent) combined with an ACE Excel 3 C18 column (100 mm × 2.1 mm, 3.0 μm), an autosampler, a quaternary pump, and a vacuum degasser. The separation conditions were as follows: flow rate, 350 μL/min; column temperature, 35 ℃; injection volume, 3 μL; sampler tray, 4 ℃; mobile phase A (water + 0.1% formic acid); and mobile phase B (acetonitrile). The gradient elution programs were as folows: 0-1.5 min, 2% B; 1.5-4.0 min, 2-20% B; 4.0-9.0 min, 20-60% B; 9.0-18.0 min, 60-98% B; 18.0-19.0 min, 98% B; 19.0-19.5 min, 98-2% B; and 19.5-21.5 min, 2% B in the positive ion mode; 0-1.5 min, 2% B; 1.5-3.0 min, 2-25% B; 3.0-7.0 min, 25-45% B; 7.0-8.0 min, 45-60% B; 8.0-15.0 min, 60-98% B; 15.0-16.0 min, 98% B; 16.0-17.5 min, 98-2% B; and 17.5-19.0 min, 2% B in the negative ion mode.

MS was performed on an Agilent 6530 Q-TOF mass spectrometer (Agilent) coupled with an electrospray ionization (ESI) source, capable of detection in both negative and positive ion modes. MassHunter Workstation software (Agilent) was employed for system operation. The operating conditions of mass spectrometer were as follows: capillary temperature, 320 ℃; capillary voltage, 4,000 V for positive ion mode; 3,500 V for negative ion mode; nebulizer, 35 psig; sheath gas flow rate, 12 L/min; collision energy, 35 eV; drying gas temperature, 300 ℃; and drying gas flow rate, 6 L/min. The mass range was set from 50 to 1100 Da in full scan mode.

MS raw data (raw) files were converted to the mzML format using ProteoWizard and processed with the R package XCMS (version 3.2), including retention time alignment, peak detection, and peak matching. Subsequently, normalization to an internal standard for each sample was performed, and missing values were replaced by half the minimum value found in the dataset by default. The preprocessing results generated a data matrix consisting of retention time (RT), mass-to-charge ratio (m/z) values, and peak intensity. OSI-SMMS (version 1.0. Dalian Chem Data Solution Information Technology Co., Ltd.) was utilized for peak annotation after data processing using an in-house MS/MS database.

## Reference

1. Liu, Xue, Shiyao Cui, Caiyun Dan, Wenle Li, Hongqing Xie, Conghui Li, Liangen Shi. 2022. “Phellinus baumii Polyphenol: A Potential Therapeutic Candidate against Lung Cancer Cells.” *International Journal of Molecular Sciences* 23: 16141. https://doi.org/10.3390/ijms232416141

2. Chamorro, S., A. Viveros, I. Alvarez, E. Vega, A. Brenes. 2012. “Changes in polyphenol and polysaccharide content of grape seed extract and grape pomace after enzymatic treatment.” *Food Chemistry* 133: 308–314. https://doi.org/10.1016/j.foodchem.2012.01.031

3. Li, Yougui, Wenyi Xu, Fang Zhang, Shi Zhong, Yuqing Sun, Jinxi Huo, Jianxun Zhu, Chongming Wu, Chaysavanh Manichanh. 2020. “The gut microbiota-produced indole-3-propionic acid confers the antihyperlipidemic effect of mulberry-derived 1-deoxynojirimycin.” *mSystems* 5: e00313–00320. https://doi.org/10.1128/mSystems.00313-20

## Supplementary figures

**
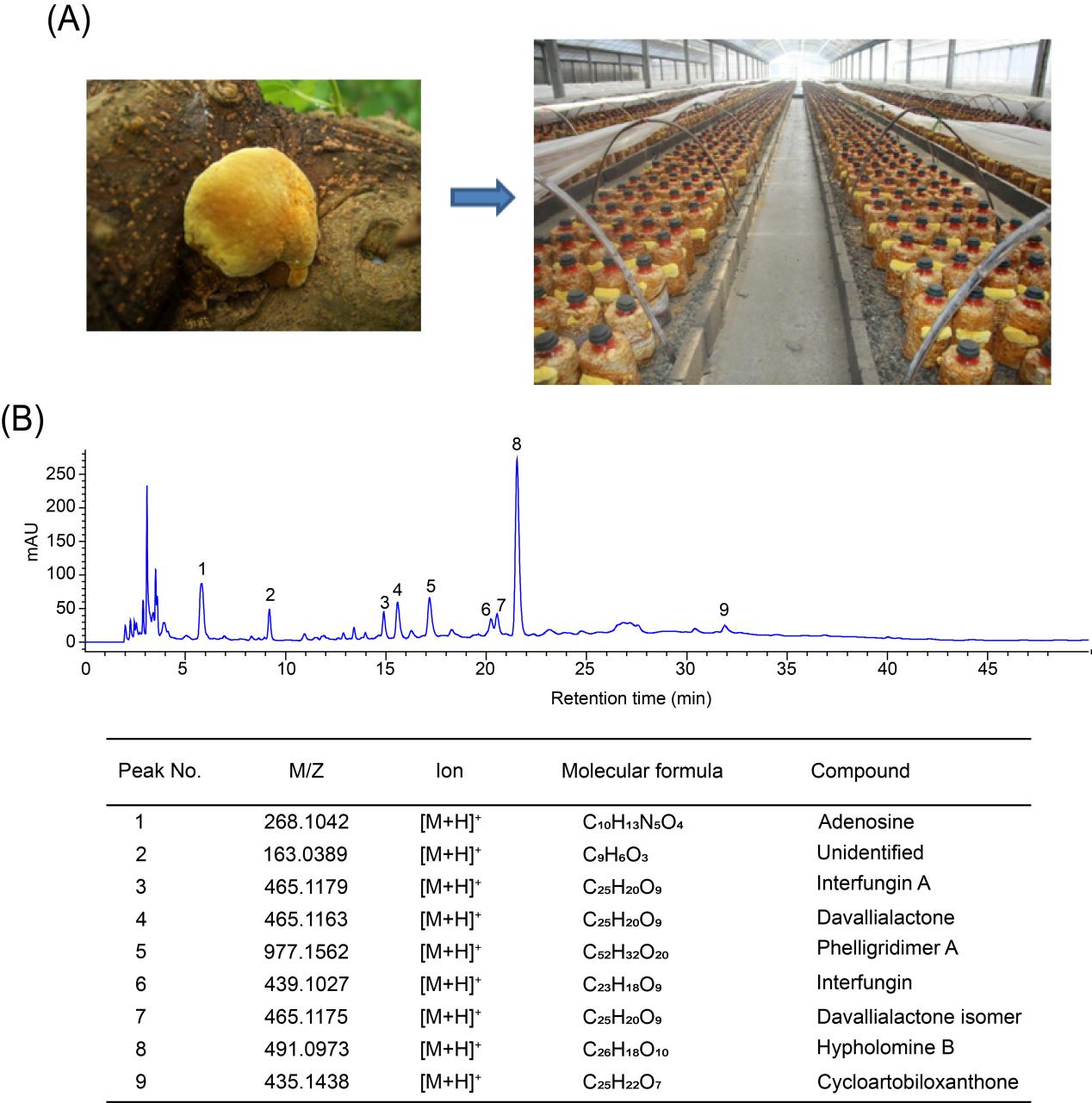
**

**Figure S1. Chemical profile of the SH extract by HPLC-TOF/MS analysis.**

**(A)** From natural growth to industrial production of SH. **(B)** The identification of SH’s chemical compounds by HPLC-TOF/MS.


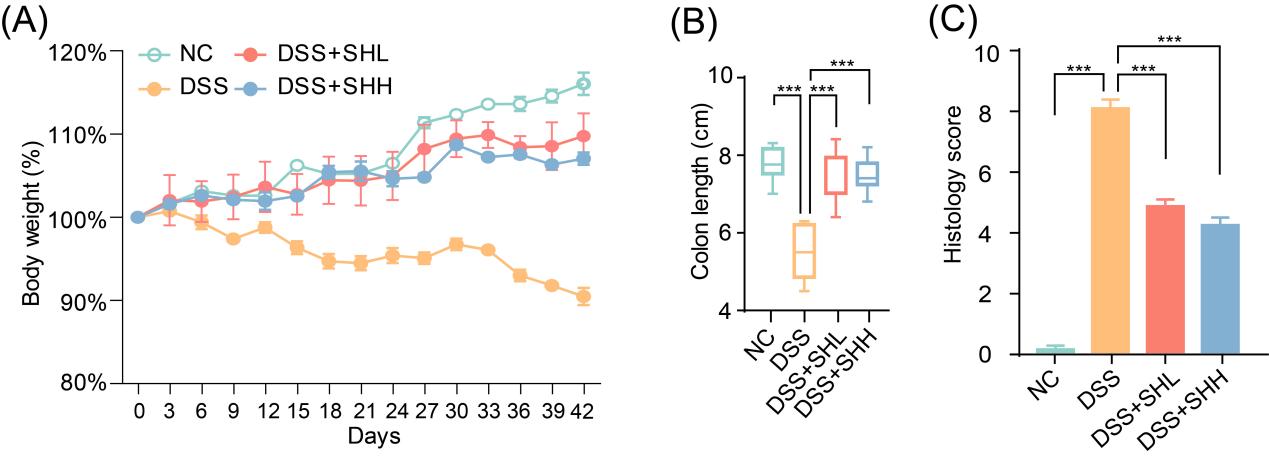


**Figure S2. The anticolitic effect of SH in DSS-induced mice.**

**(A)** Percentage change in body weight (%) of C57BL/6 colitic mice with SH treatment from 0 to 42 days. **(B)** Colon length (cm) of each group of mice. **(C)** Histology score of each group of mice. Statistical analysis was done by one-way analysis of variance (ANOVA) followed by Dunnett’s test. Data are shown as means ± SEMs (n = 8). ^*^*p* < 0.05, ^**^*p* < 0.01, ^***^*p* < 0.001.


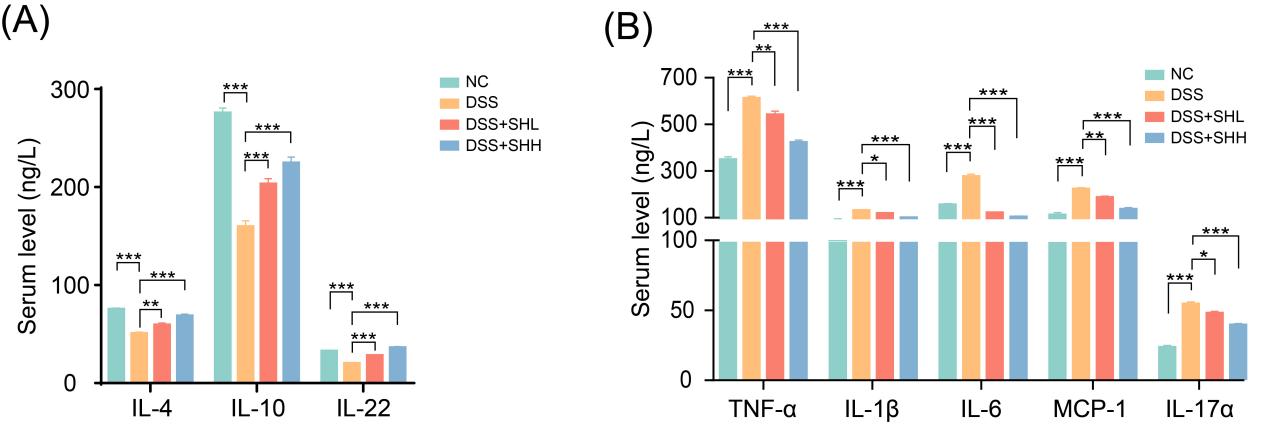


**Figure S3. The anti-inflammatory effect of SH in DSS-induced mice.**

**(A)** Serum levels of anti-inflammatory cytokines (IL-4, IL-10 and IL-22). **(B)** Serum levels of pro-inflammatory cytokines (TNF-α, IL-1β, IL-6, MCP-1 and IL-17α). Statistical analysis was done by one-way analysis of variance (ANOVA) followed by Dunnett’s test. Data are shown as means ± SEMs (n = 8). ^*^*p* < 0.05, ^**^*p* < 0.01, ^***^*p* < 0.001.


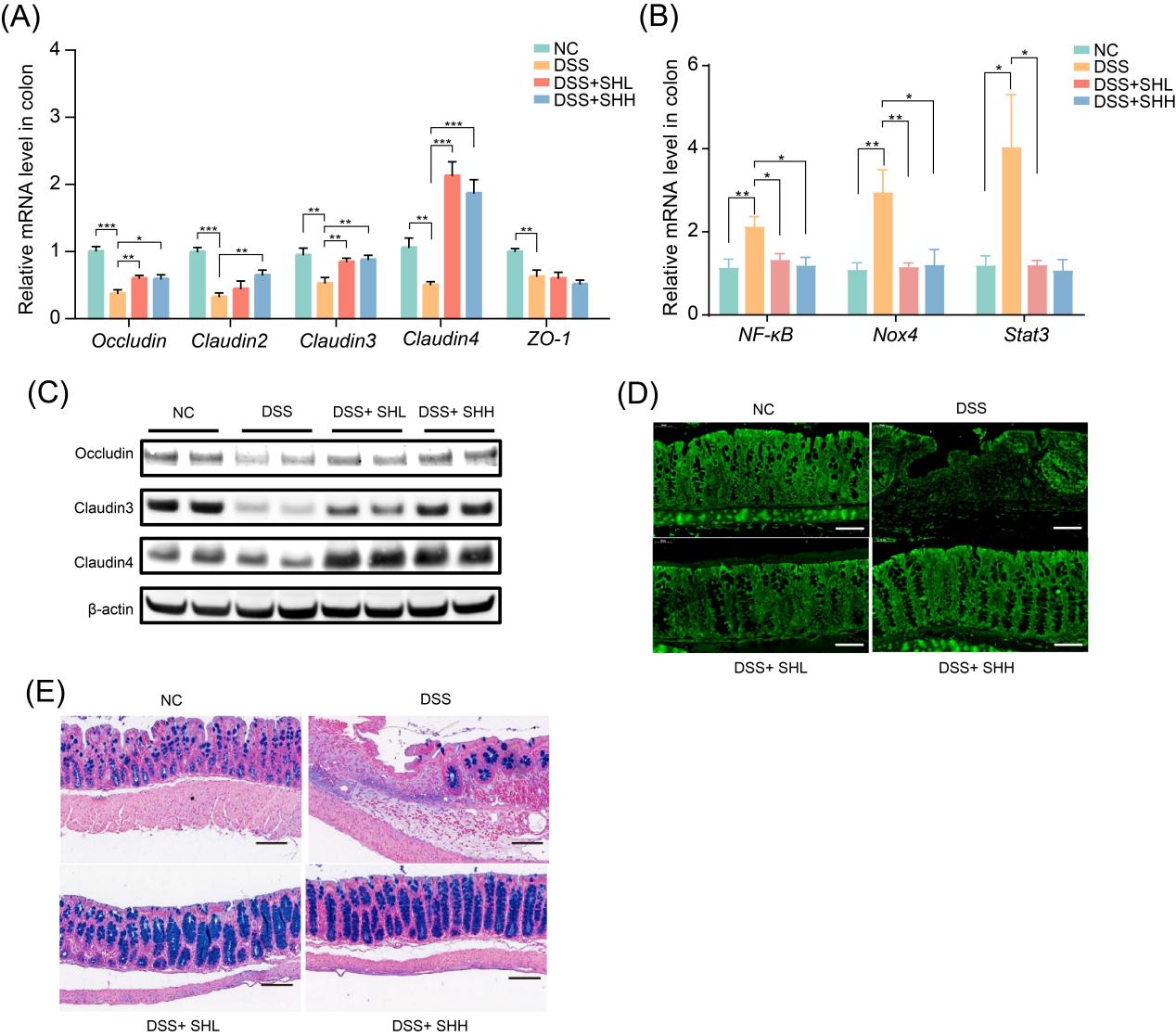


**Figure S4. The protective effect of SH on the intestinal mucosal barrier in DSS-induced mice.**

**(A)** The relative transcriptional levels of *Occludin*, *Claudin-2*, *Claudin-3*, *Claudin-4* and *ZO-1* in colon tissues. **(B)** The relative transcriptional levels of *NF-κB*, *Nox4* and *Stat3* in colon tissues. (**C**) Western blotting analysis of Occludin, Claudin-3 and Claudin-4. (**D**) Representative immunofluorescence images of tight junction structures using antibody against Claudin-4 (scale bars = 50 µm). (**E**) Representative images of colon sections stained with Alcian blue to indicate goblet cells (scale bars = 50 µm). Statistical analysis was done by one-way analysis of variance (ANOVA) followed by Dunnett’s test. Data are shown as means ± SEMs (n = 8). ^*^*p* < 0.05, ^**^*p* < 0.01, ^***^*p* < 0.001.


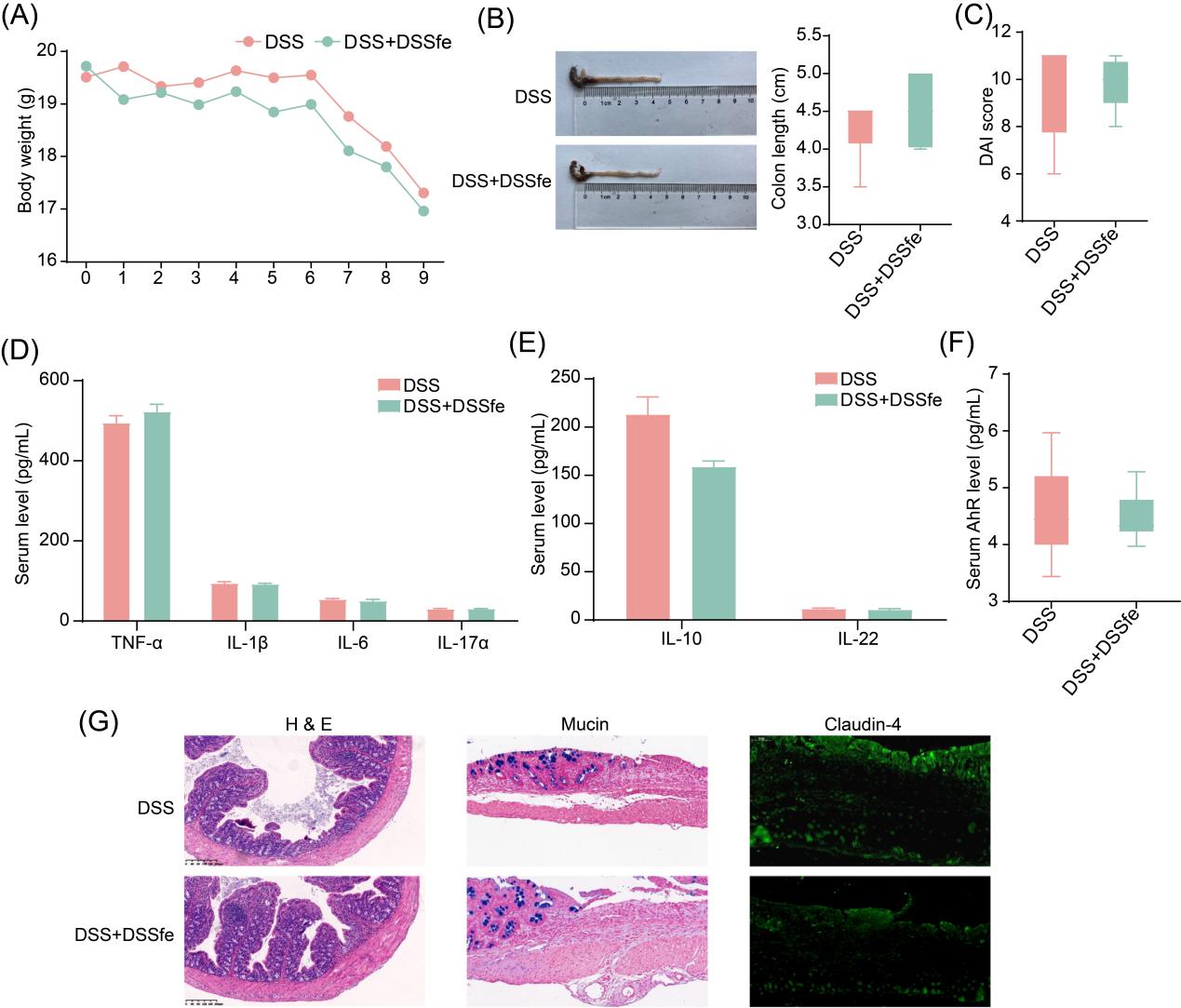


**Figure S5. The effect of DSS-modulated changes in the gut microbiota on colitis symptoms in DSS-induced mice.**

(**A**) The body weight of colitic mice. **(B)** Representative images of the colonic tissue and colon length (cm). **(C)** DAI score. **(D)** Serum levels of pro-inflammatory cytokines (TNF-α, IL-1β, IL-6 and IL-17α). **(E)** Serum levels of anti-inflammatory cytokines (IL-10 and IL-22). **(F)** Serum levels of AhR. **(G)** Representative images of colon sections stained with hematoxylin and eosin (H&E) (scale bars = 200 µm), representative images of colon sections stained with Alcian blue to indicate goblet cells (scale bars = 50 µm), and representative immunofluorescence images of tight junction structures using antibody against Claudin-4 (scale bars = 50 µm). Statistical analysis was done by one-way analysis of variance (ANOVA) followed by Dunnett’s test. Data are shown as means ± SEMs (n = 8).


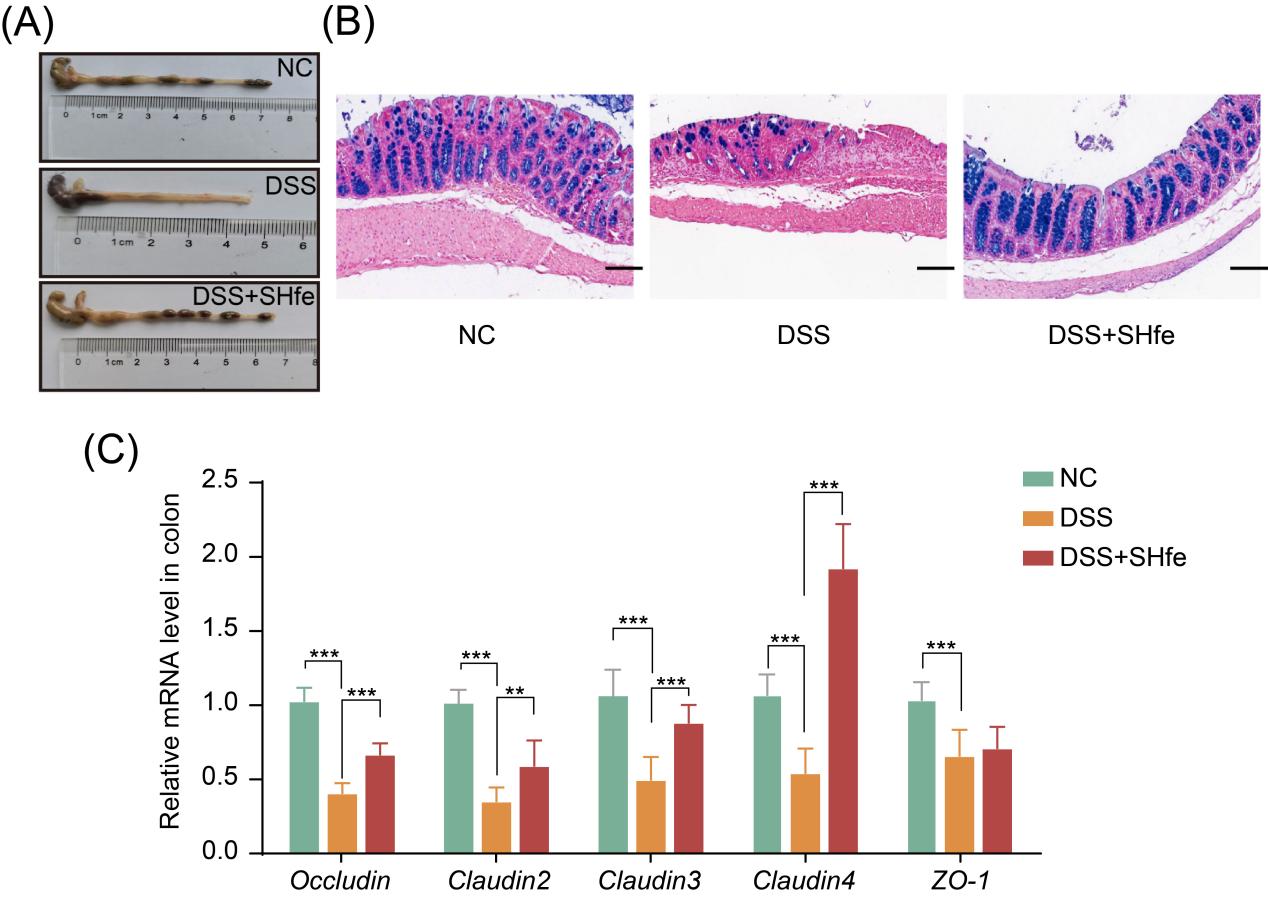


**Figure S6. The protective effect of the SH-modulated gut microbiota on colonic tissue in DSS-induced mice.**

**(A)** Representative images of the colonic tissue. **(B)** Representative images of colon sections stained with Alcian blue to indicate goblet cells (scale bars = 50 µm). **(C)** The relative transcriptional levels of *Occludin*, *Claudin-2*, *Claudin-3*, *Claudin-4* and *ZO-1*. Statistical analysis was done by one-way analysis of variance (ANOVA) followed by Dunnett’s test. Data are shown as means ± SEMs (n = 8). ^*^*p* < 0.05, ^**^*p* < 0.01, ^***^*p* < 0.001.


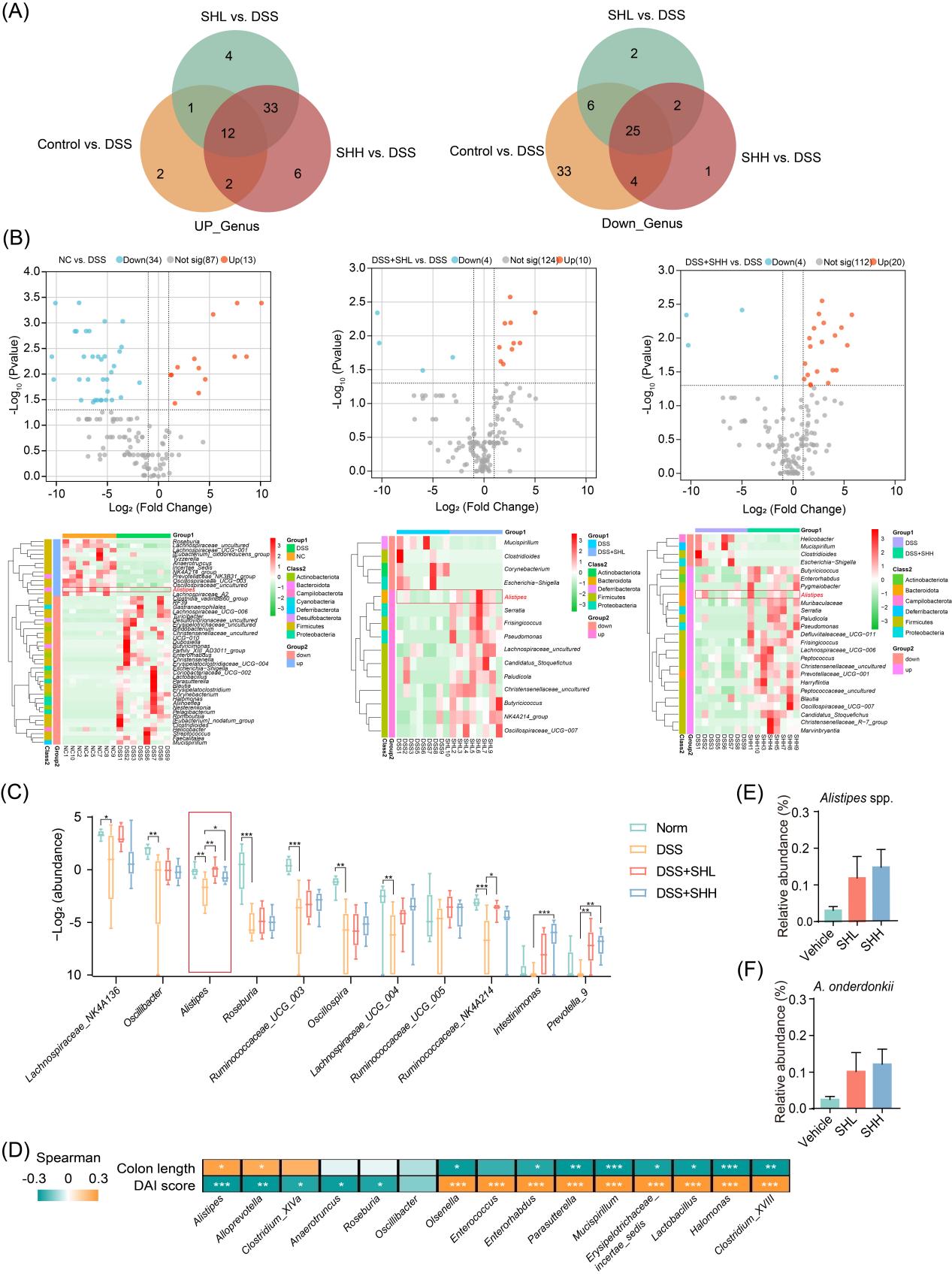


**Figure S7. *Alistipes* is a SH-enriched genus involved in the colitis-ameliorating effect of SH.**

**(A)** Venn diagram showing the overlapping of upregulated (UP) genera and downregulated genera among control vs. DSS, SHL vs. DSS and SHH vs. DSS. **(B)** Volcano plot and heatmap comparing up-regulated and down-regulated genera across different groups. **(C)** Log2-transformed relative abundance of eleven significant altered genera. (D) Correlation analysis between differential gut microbiota and DAI score, colon length based on spearman correlation coefficient. **(E)** Relative abundance of *Alistipes* spp. and **(F)** *A. onderdonkii* in vehicle, SHL and SHH group. Statistical analysis was done by Wilcox nonparametric test. Data are shown as means ± SEMs (n = 8). ^*^*p* < 0.05, ^**^*p* < 0.01, ^***^*p* < 0.001.


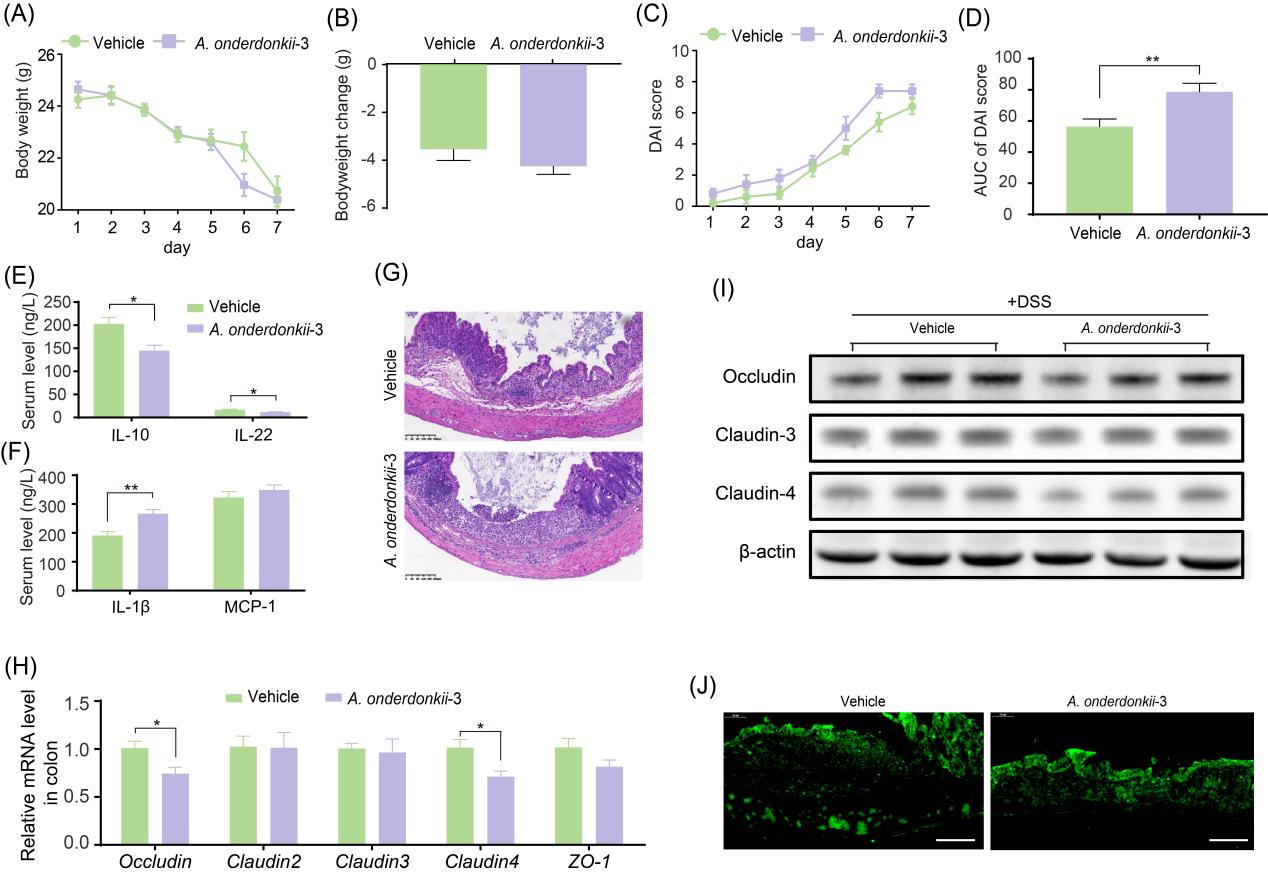


**Figure S8. S3 One strain of *Alistipes onderdonkii* barely exerts its anti-colitis effect**.

**(A-B)** Body weight curve from 1 to 7 days and change of body weight (g). **(C-D)** DAI score and AUC of DAI score. **(E-F)** Serum levels of anti-inflammatory cytokines (IL-10 and IL-22) and pro-inflammatory cytokines (IL-1β and MCP-1). **(G)** Representative images of the colonic sections stained with H&E (scale bars = 200 µm). **(H)** Relative mRNA levels of *Occludin*, *Claudin-2*, *Claudin-3*, *Claudin-4* and *ZO-1* in colon. **(I)** Western blot of Occludin, Claudin-3 and Claudin-4 proteins. **(J)** Immunofluorescence imaging of the structure of tight junction using anti-Claudin-4 antibody (scale bars = 50 µm). Statistical analysis was done by one-way analysis of variance (ANOVA) followed by Dunnett’s test. Data are shown as means ± SEMs (n = 8). ^*^*p* < 0.05, ^**^*p* < 0.01, ^***^*p* < 0.001.


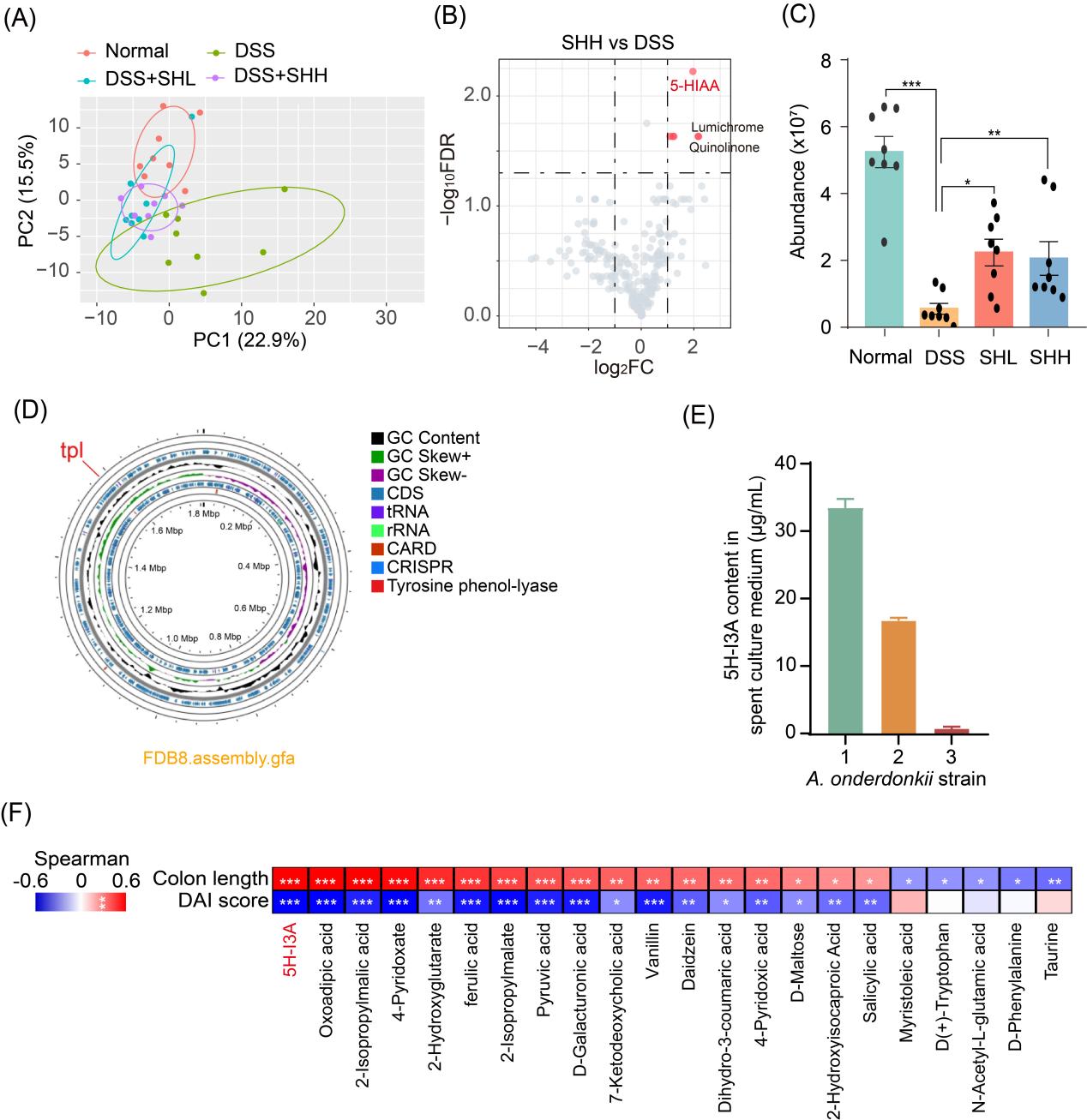


**Figure S9. 5-Hydroxyindole-3-acetic acid (5HIAA) is an SH-enriched gut microbial metabolite that mediates the anti-colitis effect of SH**.

**(A)** Principal component analysis (PCA) for metabolites profile of mice cecal materials. **(B)** Volcano plot comparing up-regulated metabolites across SHH and DSS group. **(C)** The abundance of 5HIAA among normal, DSS, SHL and SHH group. **(D)** The genome of strain FDB8. **(E)** The level of 5HIAA in the spent culture supernatants of *A. onderdonkii* using high-performance liquid chromatography (HPLC). **(F)** Correlation analysis between differential metabolites and DAI score, colon length based on Spearman correlation coefficient. Statistical analysis was done by one-way analysis of variance (ANOVA) followed by Dunnett’s test. Data are shown as means ± SEMs (n = 8). ^*^*p* < 0.05, ^**^*p* < 0.01. HPLC, high-performance liquid chromatography; 5HIAA, 5-Hydroxyindole-3-acetic acid.


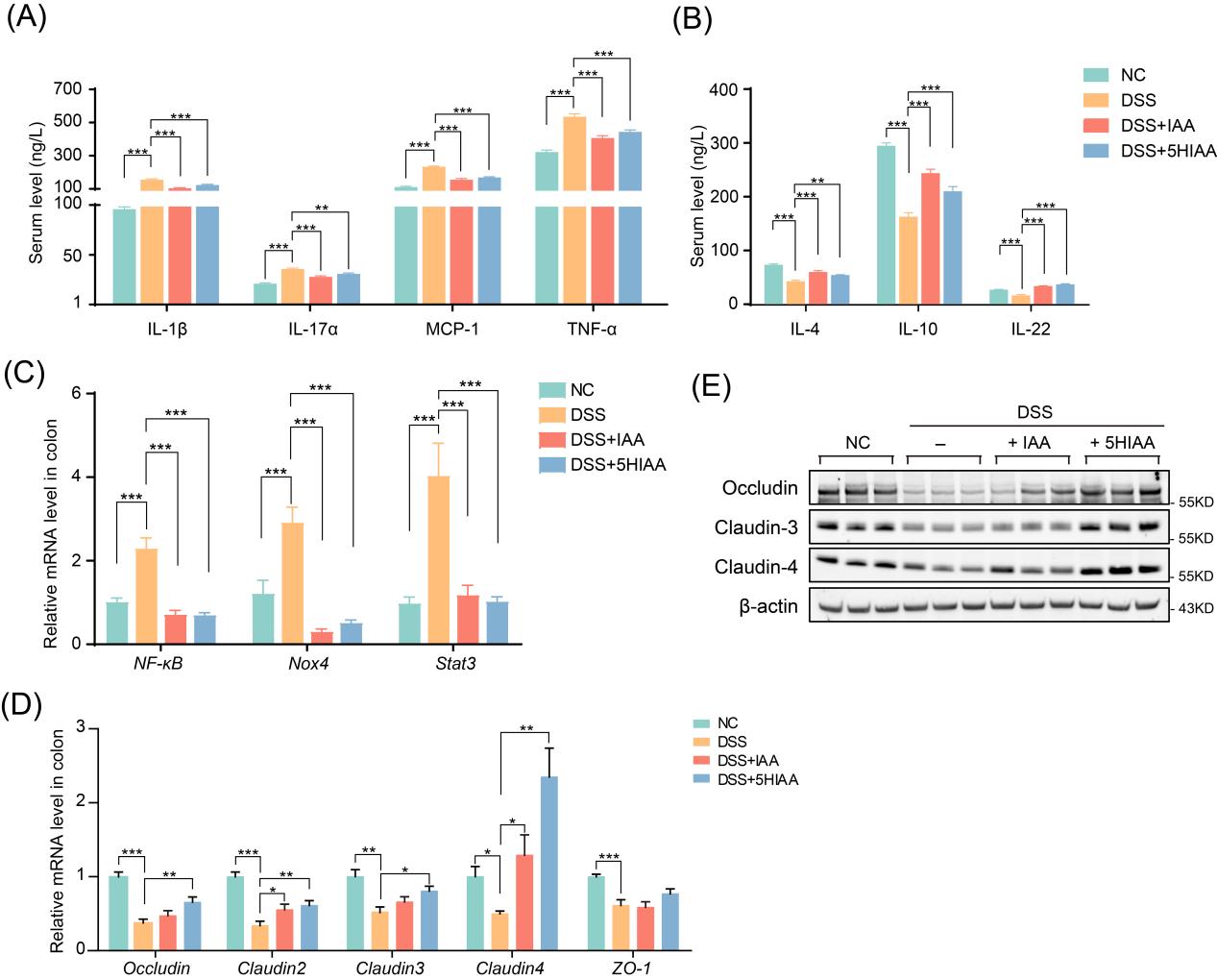


**Figure S10. Treatment with 5HIAA alleviates DSS-induced inflammation and intestinal mucosal barrier injury in C57BL/6 mice**.

**(A)** Serum levels of pro-inflammatory cytokines (IL-1β , IL-17α, MCP-1 and TNF-α). **(B)** Serum levels of anti-inflammatory cytokines (IL-4, IL-10 and IL-22). **(C)** Relative mRNA levels of *NF-κB*, *Nox4* and *Stat3* in colon tissues. **(D)** Relative mRNA levels of *Occludin*, *Claudin-2*, *Claudin-3*, *Claudin-4* and *ZO-1* in colon tissues. **(E)** Western blot of Occludin, Claudin-3 and Claudin-4 proteins. Statistical analysis was done by one-way analysis of variance (ANOVA) followed by Dunnett’s test. Data are shown as means ± SEMs (n = 8). ^*^*p* < 0.05, ^**^*p* < 0.01, ^***^*p* < 0.001. IAA, indole-3-acetic acid.

**
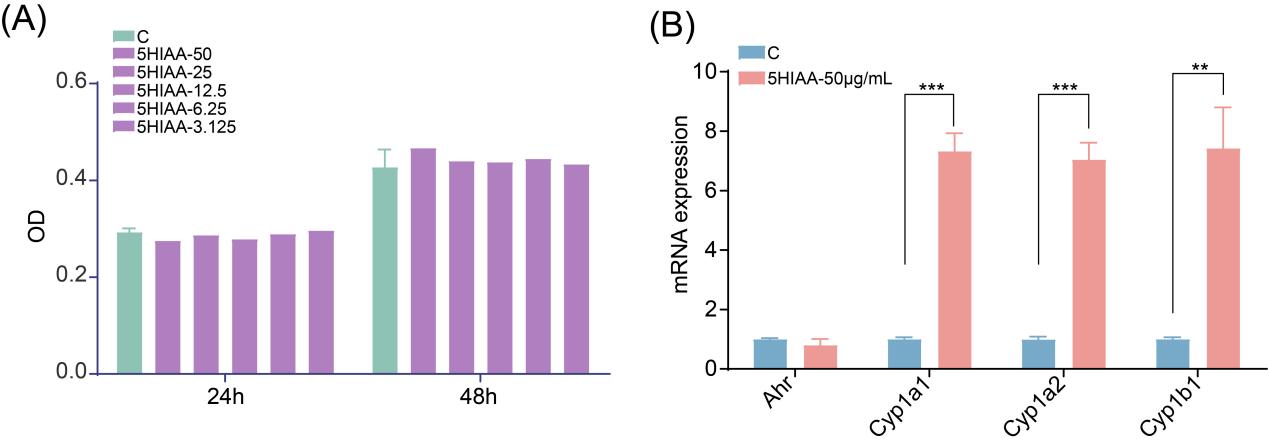
**

**Figure S11. Treatment with 5HIAA activates the AhR signaling pathway in Caco2 cells**. **(A)** CCK-8 assay dispalys the cytotoxicity of five different 5HIAA concentrations in cell proliferation. **(B)** The expression level of *Ahr*, *Cyp1a1*, *Cyp1a2* and *Cyp1b1*. ^**^*p* < 0.01, ^***^*p* < 0.001.

## Supplementary tables

**Table S1. Characterization of the chemical constituents of SH by HPLC-TOF/MS**

| Peak NO. | Formula | Identification | 2D structure |
| --- | --- | --- | --- |
| 1 | C_10_H_13_N_5_O_4_ | Adenosine |  |
| 2 | C_9_H_6_O_3_ | unidentified | unidentified |
| 3 | C_25_H_20_O_9_ | Interfungin A |  |
| 4 | C_25_H_20_O_9_ | Davallialactone |  |
| 5 | C_52_H_32_O_20_ | Phelligridimer A |  |
| 6 | C_23_H_18_O_9_ | Interfungin C |  |
| 7 | C_25_H_20_O_9_ | Davallialactone isomer | unidentified |
| 8 | C_26_H_18_O_10_ | Hypholomine B |  |
| 9 | C_25_H_22_O_7_ | Cycloartobiloxanthone |  |

**Table S2. qRT-PCR primers used in this work.**

| Gene | Forward primer(5’- 3’) | Reverse primer (5’- 3’) |
| --- | --- | --- |
| All bacteria | TCCTACGGGAGGCAGCAGTG | TTACCGCGGCTGCTGGCACG |
| *Alistipes spp.* | TTAGAGATGGGCATGCGTTGT | TGAATCCTCCGTATT |
| *Alistipes onderdonkii* | ACAAGCACCTGGAACTCTCG | CTTGCCTTCGGAGTGGATGT |
| Occludin | CTCTTTGGAGGAAGCCTAA | GAAGCGATGAAGCAGAAG |
| Claudin-3 | ACCAACTGCGTACAAGACGAG | CGGGCACCAACGGGTTATAG |
| Claudin-4 | GGAGGGCCTCTGGATGAACT | GATGCTGATGACCATAAGGGC |
| CYP1A1 | GGAAGTGGAAGGGCATAGGCA | TCCAAGGCAGAATACGGTGAC |
| CYP1A2 | GCTTCTCCATAGCCTCGGAC | CTGGCTGACTGGTTCGAAGT |
| CYP1B1 | CTGGACAAGGACGGCTTCAT | ACAGTTCCTCACCGATGCAC |
| TNF-α | ATCCGCGACGTGGAACTG | ACCGCCTGGAGTTCTGGAA |
| IL-1β | TGTGTTTTCCTCCTTGCCTCTGAT | TGCTGCCTAATGTCCCCTTGAAT |
| IL-6 | GAGGATACCACTCCCAACAGACC | AAGTGCATCATCGTTGTTCATACA |
| IL-17α | TTTAACTCCCTTGGCGCAAAA | CTTTCCCTCCGCATTGACAC |
| MCP-1 | TAAAAACCTGGATCGGAACCAAA | GCATTAGCTTCAGATTTACGGGT |
| IL-10 | CTTACTGACTGGCATGAGGATCA | GCAGCTCTAGGAGCATGTGG |
| IL-22 | ATGAGTTTTTCCCTTATGGGGAC | GCTGGAAGTTGGACACCTCAA |
| β-actin | CATGTACGTTGCTATCCAGGC | CTCCTTAATGTCACGCACGAT |

## West blotting original image

Fig.02 i


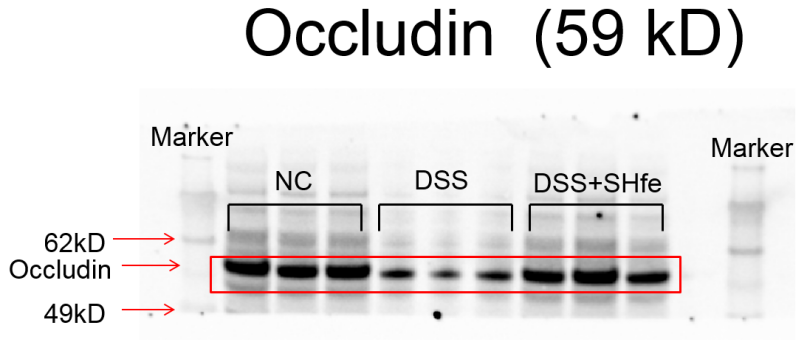


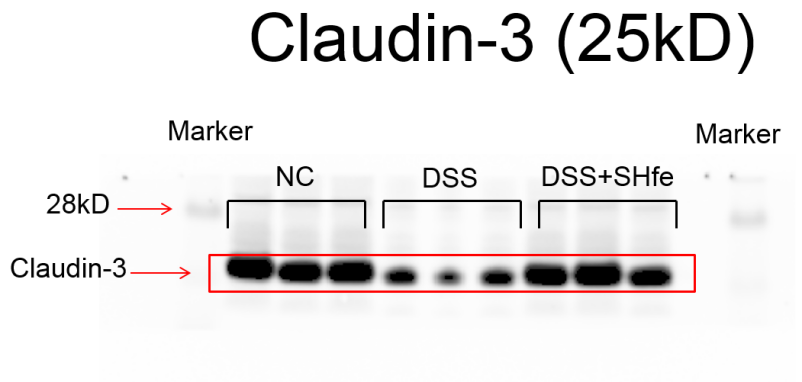


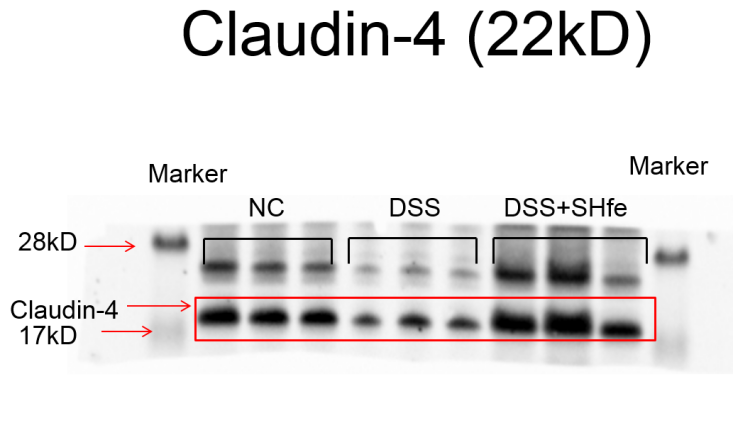


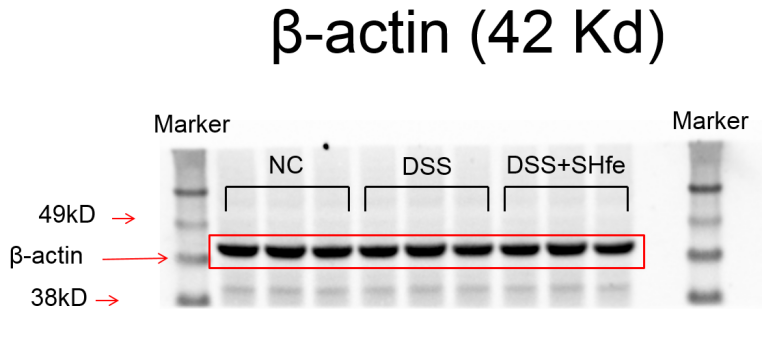


Fig.03 g


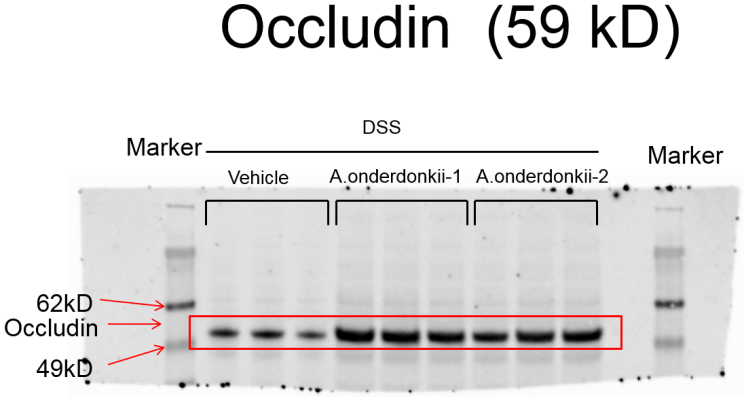

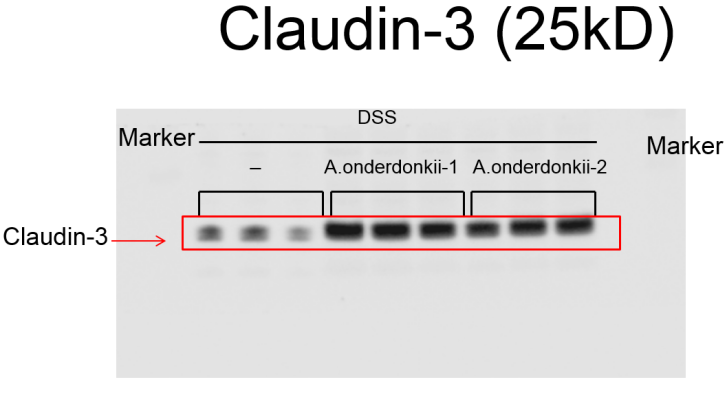

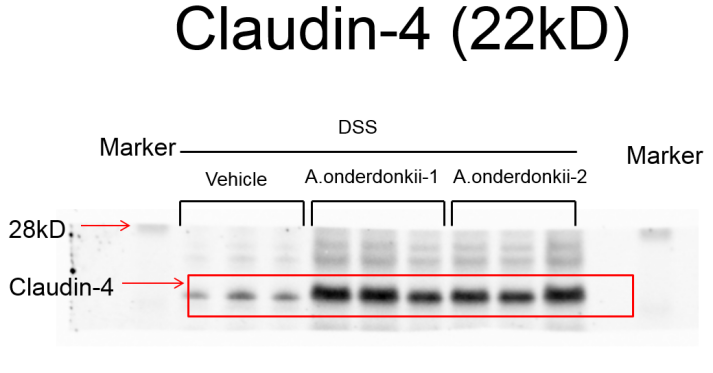

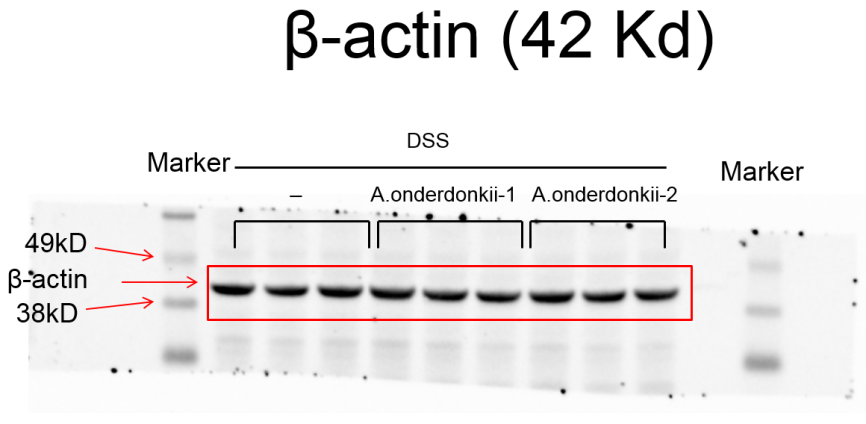


Fig.S4 b


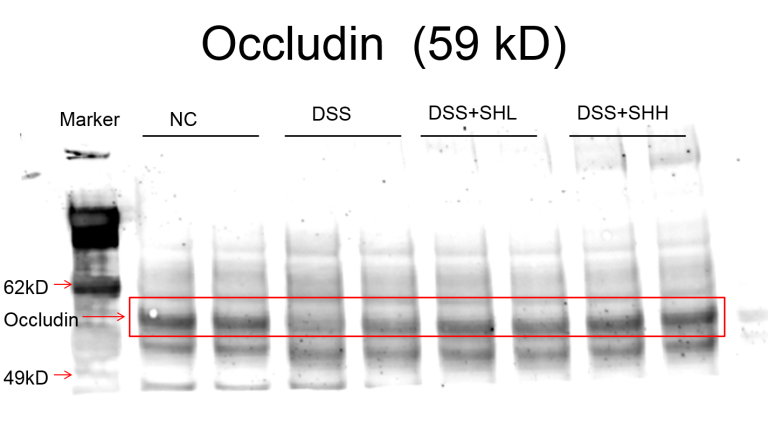


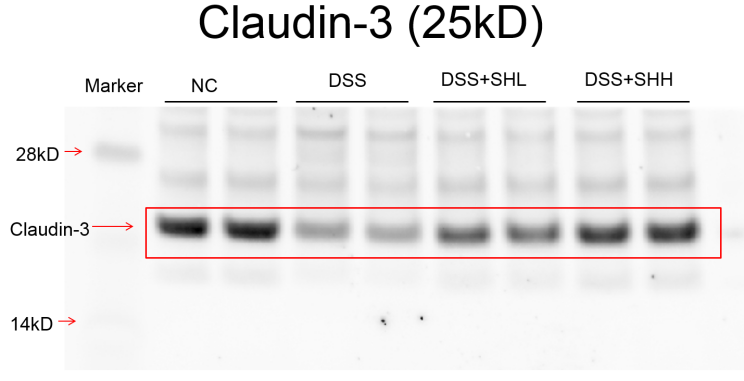


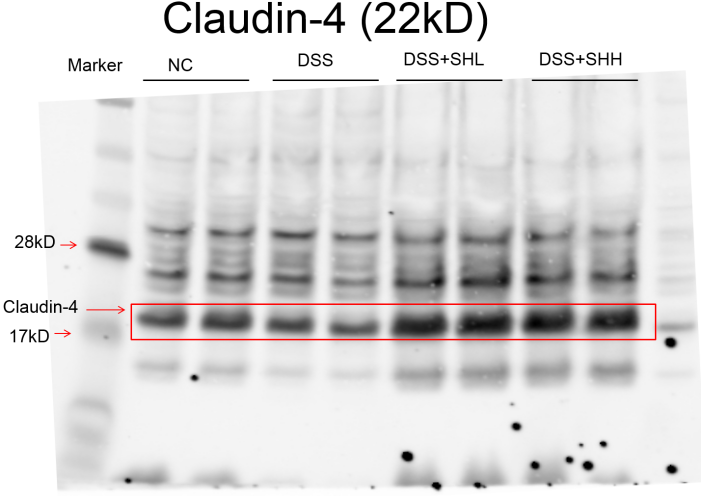


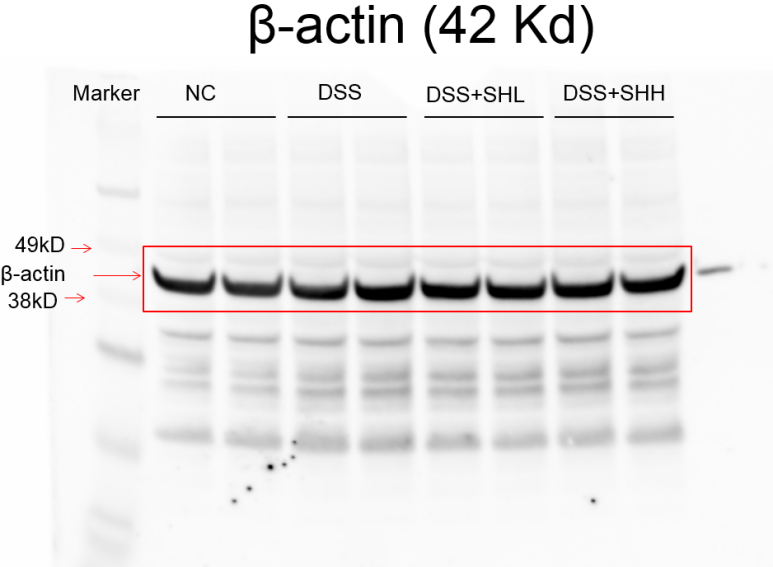


Fig.S7 i


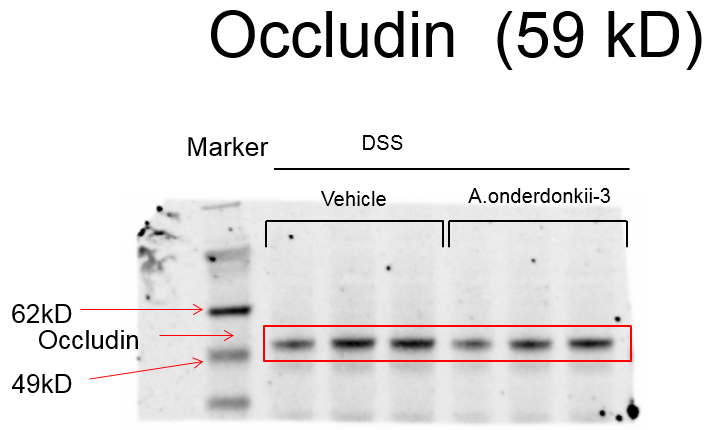


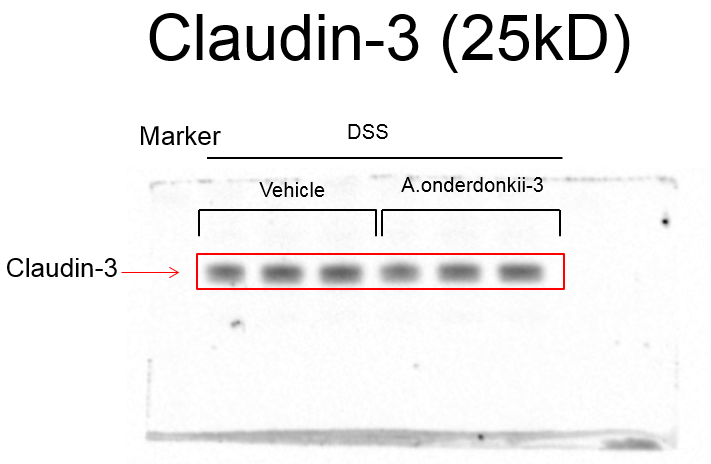


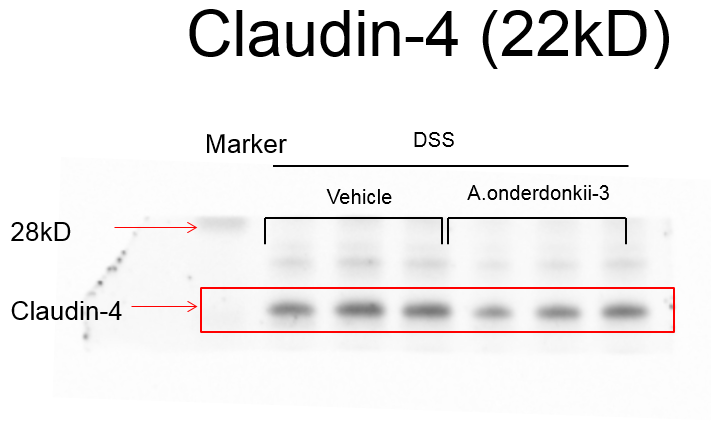


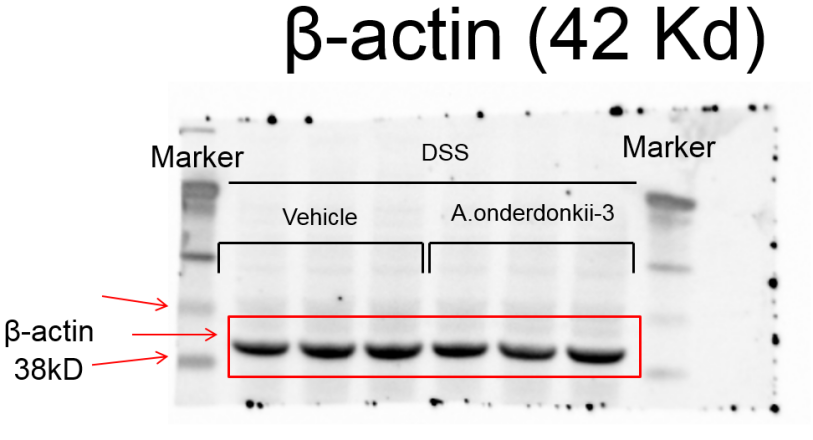


Fig.S9 d


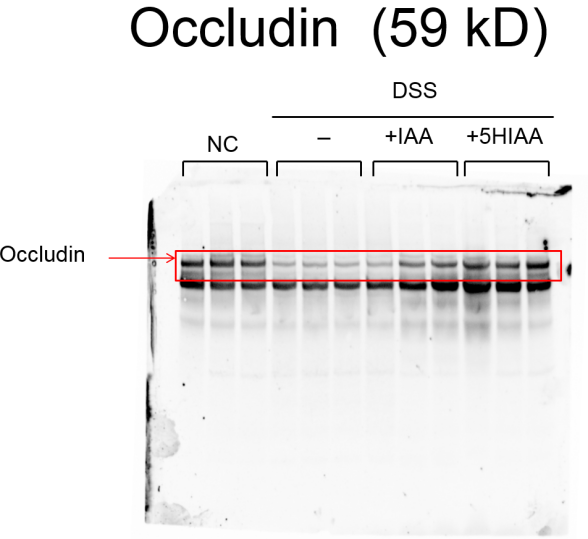


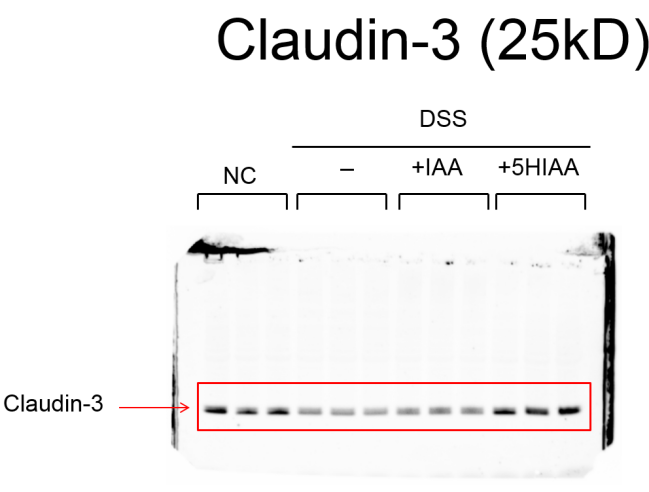


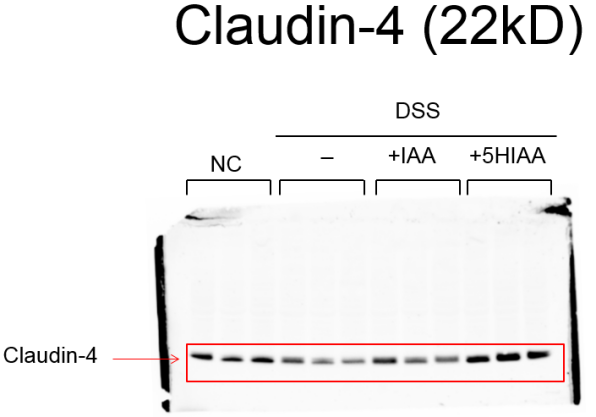


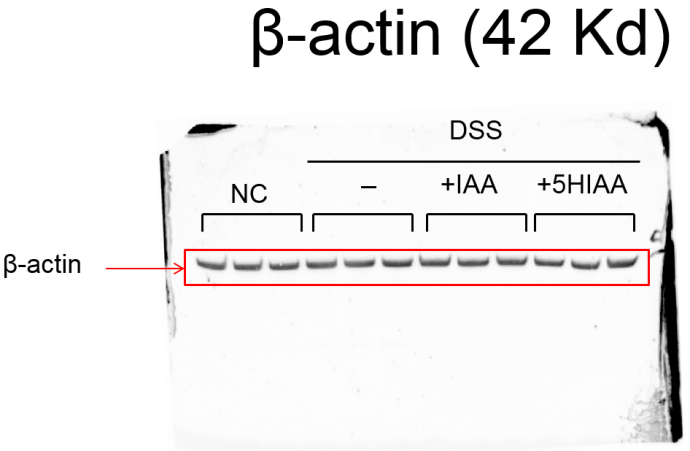

Supplement: Supplementary file 1 — Figure S1. Chemical profile of the SH extract by HPLC‐TOF/MS analysis. Figure S2. The anticolitic effect of SH in DSS‐induced mice. Figure S3. The anti‐inflammatory effect of SH in DSS‐induced mice. Figure S4. The protective effect of SH on the intestinal mucosal barrier in DSS‐induced mice. Figure S5. The effect of DSS‐modulated changes in the gut microbiota on colitis symptoms in DSS‐induced mice. Figure S6. The protective effect of the SH‐modulated gut microbiota on colonic tissue in DSS‐induced mice. Figure S7. Alistipes is a SH‐enriched genus involved in the colitis‐ameliorating effect of SH. Figure S8. S3 One strain of Alistipes onderdonkii barely exerts its anticolitis effect. Figure S9. 5‐Hydroxyindole‐3‐acetic acid (5HIAA) is an SH‐enriched gut microbial metabolite that mediates the anticolitis effect of SH. Figure S10. Treatment with 5HIAA alleviates DSS‐induced inflammation and intestinal mucosal barrier injury in C57BL/6 mice. Figure S11. Treatment with 5HIAA activates the AhR signaling pathway in Caco2 cells. Table S1. Characterization of the chemical constituents of SH by HPLC‐TOF/MS. Table S2. qRT‒PCR primers used in this work. [file IMT2-3-e180-s001.docx]
